# Circulating Biomarkers Associated with the Diagnosis and Prognosis of B-Cell Progenitor Acute Lymphoblastic Leukemia

**DOI:** 10.3390/cancers15164186

**Published:** 2023-08-20

**Authors:** Claudia Daniela Álvarez-Zúñiga, Idalia Garza-Veloz, Jacqueline Martínez-Rendón, Misael Ureño-Segura, Iván Delgado-Enciso, Margarita L. Martinez-Fierro

**Affiliations:** 1 Molecular Medicine Laboratory, Unidad Académica de Medicina Humana y C.S, Universidad Autónoma de Zacatecas, Zacatecas 98160, Mexico; cldaalzu30798@gmail.com (C.D.Á.-Z.); idaliagv@uaz.edu.mx (I.G.-V.); jamare85@gmail.com (J.M.-R.); 2 Hematology Service, Hospital General Zacatecas “Luz González Cosío”, Servicios de Salud de Zacatecas, Zacatecas 98160, Mexico; misa19250@gmail.com; 3 Cancerology State Institute, Colima State Health Services, Colima 28085, Mexico; ivan_delgado_enciso@ucol.mx; 4 School of Medicine, University of Colima, Colima 28040, Mexico

**Keywords:** biomarkers, circulating, acute lymphoblastic leukemia, BCP-ALL, diagnosis, prognosis

## Abstract

**Simple Summary:**

Acute lymphoblastic leukemia (ALL) is a hematological disease characterized by the malfunction of the hematopoietic system. This process is generated and perpetuated by several mechanisms that provide the disease with part of its malignancy potential and genetic and cytological characteristics. Disease pathological features are used for diagnosis and prognosis; however, most of them are obtained by a bone marrow aspirate, so it is crucial to find other less invasive methods to establish or complement the diagnosis, prognosis, and follow-up of the disease. A cancer biomarker is any measurable indicator demonstrating the presence of malignancy, tumor behavior, prognosis, or treatment responses. This review will summarize circulating diagnostic and prognosis biomarkers for B-cell progenitor ALL.

**Abstract:**

Acute lymphoblastic leukemia (ALL) is a hematological disease characterized by the dysfunction of the hematopoietic system that leads to arrest at a specific stage of stem cells development, suppressing the average production of cellular hematologic components. BCP-ALL is a neoplasm of the B-cell lineage progenitor. BCP-ALL is caused and perpetuated by several mechanisms that provide the disease with its tumor potential and genetic and cytological characteristics. These pathological features are used for diagnosis and the prognostication of BCP-ALL. However, most of these paraclinical tools can only be obtained by bone marrow aspiration, which, as it is an invasive study, can delay the diagnosis and follow-up of the disease, in addition to the anesthetic risk it entails for pediatric patients. For this reason, it is crucial to find noninvasive and accessible ways to supply information concerning diagnosis, prognosis, and the monitoring of the disease, such as circulating biomarkers. In oncology, a biomarker is any measurable indicator that demonstrates the presence of malignancy, tumoral behavior, prognosis, or responses to treatments. This review summarizes circulating molecules associated with BCP-ALL with potential diagnostic value, classificatory capacity during monitoring specific clinic features of the disease, and/or capacity to identify each BCP-ALL stage regarding its evolution and outcome of the patients with BCP-ALL. In the same way, we provide and classify biomarkers that may be used in further studies focused on clinical approaches or therapeutic target identification for BCP-ALL.

## 1. Introduction

Leukemias are a group of hematological diseases characterized by an abnormal cell population suppressing the average production of cellular components of the hematopoietic system [1]. There are four main subtypes of leukemia that have been identified based on their evolution time and hematological lineage: acute lymphoblastic leukemia (ALL), chronic lymphocytic leukemia (CLL), acute myelogenous leukemia (AML), and chronic myelogenous leukemia (CML) [2]. ALL and CLL are the most common leukemia types in children and adults, respectively [3,4].

ALL is a malignant disorder that occurs when typical lymphoid cell development malfunctions due to arrest at a specific stage of development; the name and classification and based on the dysfunctional stage [5]. ALL can develop from primitive precursor cells with multilineage potential from the two types of lymphoblasts: B or T. These cells proliferate uncontrollably in bone marrow (BM) and peripheral blood (PB) [1,6,7,8] and interfere with the functions of normal blood cells [8,9]. Of the two kinds of lymphoblasts, it is more common for ALL to originate from B-cell progenitor (BCP-ALL), representing about 80% of cases; the remaining 20% originate from T-cell progenitors (TCP-ALL) [10]. 

According to the World Health Organization (WHO), BCP-ALL is defined as a neoplasm of precursor lymphoid cells committed to the B cell lineage, typically composed of small-to-medium-sized blast cells with scant cytoplasm, moderately condensed to dispersed chromatin, and inconspicuous nucleoli [5]. BCP-ALL diagnosis involves clinical and laboratory tests. BCP-ALL symptoms indicate blast infiltration in the bone marrow, lymphoid system, and extramedullary sites and may include fatigue or lethargy, constitutional symptoms, dyspnea, dizziness, infections, and easy bruising or bleeding [11]. A BM aspirate is mandatory for laboratory tests to confirm the diagnosis, demonstrating ≥ 20% bone marrow lymphoblasts [12]. In addition, clinicians can examine cell morphology, immunophenotyping, and genetics and integrate these measures according to the classification established by the WHO [5]. BCP-ALL has multiple subtypes characterized by immunophenotype B-I (pro-B), B-II (Common), B-III (pre-B), and B-IV (B—mature Burkitt type) [5]. Recently, the nomenclature of leukemia with genetic abnormalities has shifted from focusing on cytogenetic alterations to molecular events, and new genetic entities have been added. The WHO classification in 2022 is based on clinical–biological entities defined by cytogenetic alterations that confer different prognoses. The United Kingdom ALL copy-number alteration (UKALL-CNA) classification has been incorporated, which distinguishes three genetic risk groups [13].

The peak incidence of ALL is from 2 to 5 years of age. It is rare in adults, but it can be found after 50 years of age. BCP-ALL constitutes approximately 80–85% of total cases of ALL [14,15,16]. Many factors affect the prognosis of BCP-ALL, such as age, the white blood cell (WBC) count, clinical features at diagnosis, cytogenetic abnormalities, pharmacodynamic and pharmacogenetic characteristics of normal patient cells, early response to therapy, and the results of the measurements of minimal/measurable residual disease (MRD: the presence of leukemic cells at specific time points in bone marrow or even in peripheral blood circulation) [17,18,19,20,21,22]. The prognostic implication of cytogenic abnormalities relates to poorly regulated signaling pathways [5,23], which can affect leukemogenesis and directly influences ALL development [24,25].

The above-mentioned factors help determine a “risk stratification” classification for BCP-ALL. This classification determines the treatment regimen and intensity that will be settled to treat BCP-ALL. This classification has contributed to a marked improvement in the prognosis of patients with leukemia [19,26,27]. 

As mentioned before, a BM puncture (BMP)/aspirate is needed to diagnose BCP-ALL. However, because of its invasive nature, potential side effects, and expensive procedures, there is a real need for alternative measures and molecular markers for the effective diagnosis and prognosis of pediatric patients with BCP-ALL [14,28]. Suitable substitutes for BMP are molecular biomarkers found in peripheral blood; collecting blood produces minimal trauma, and biomarkers can be measured relatively easy and early in the disease [29,30,31]. Nevertheless, the mentioned replacement must fulfill the biomarker standards. 

In oncology, a biomarker is any measurable indicator that demonstrates the presence of malignancy, tumor behavior, prognosis, or responses to treatments [32]. Among the myriad of biological materials circulating in the bloodstream, the most promising biomarkers include circulating tumor cells, cell-free DNA (cf-DNA) and RNA, proteins and metabolites, and extracellular vesicles (EVs) [33]. In this review, we integrated and summarized circulating biomarkers with diagnostic and prognostic potential for BCP-ALL. We searched PubMed, using the search terms “plasma biomarker” OR “circulating biomarker” AND “acute lymphoblastic leukemia”, to identify papers published in English up to December 2022. We used and restricted the search to specific study designs: human, epidemiological studies, clinical trials, clinical practice guidelines, meta-analyses, observational studies, and systematic reviews. We also searched the reference lists from articles and reviews we identified in the search. 

## 2. Leukemogenesis and Associated Cell Processes

**Figure 1:**
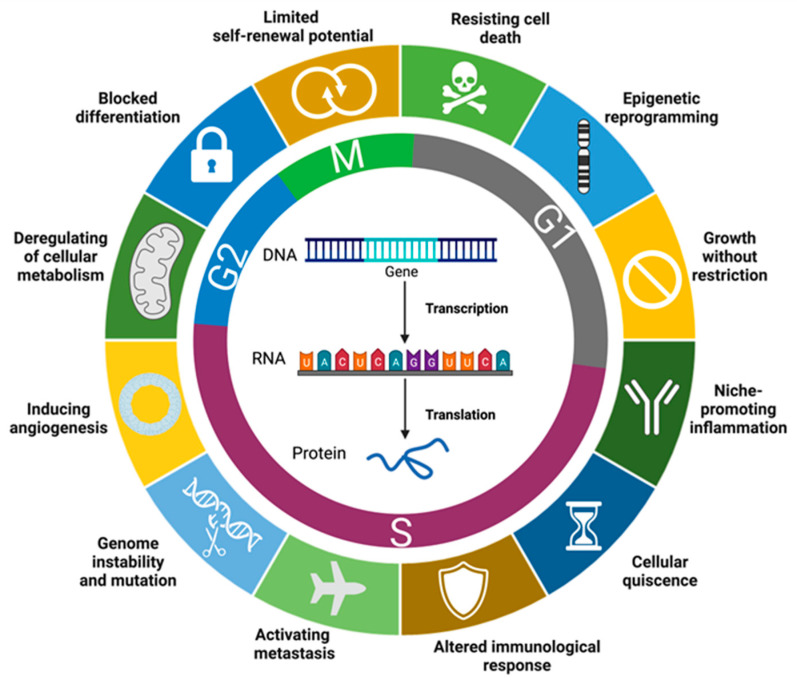
The mechanisms involved in leukemogenesis. Cell cycle dysfunction through one or several molecular mechanisms disturbs the gene regulation machinery. The level of disturbance in the machinery determines the product that will be found in the bloodstream. Adapted from [41]. 2022 Hanahan D.

Lymphoid-primed multipotent progenitors initiate B-cell development. These cells evolve into common lymphoid progenitors, which then develops into pro–B cells, then pre–B cells, and finally mature B cells [34]. The subtype of BCP-ALL is determined by the level of developmental arrest. The Leukemic Stem Cells (LSC) are malignant cells with multilineage potential; they produce the leukemic cell population. LSC block differentiation and can proliferate in PB, BM, and other organs. [1,6,7,8,35,36]. Moreover, their rapid growth leads to accumulation that may interfere with the suppression of normal hematopoiesis [8,9]. The entire process mentioned above is caused and perpetuated by common hallmarks of cancer and includes the following molecular mechanism: limited self-renewal potential, deregulating cellular metabolism, blocking differentiation, resisting cell death, genome instability and mutation, epigenetic reprogramming, growth without restriction, metastasis, inducing angiogenesis, cellular quiescence, altered immunologic response, and niche-promoting inflammation (Figure 1) [35,36,37,38,39,40,41]. 

**Figure 2:**
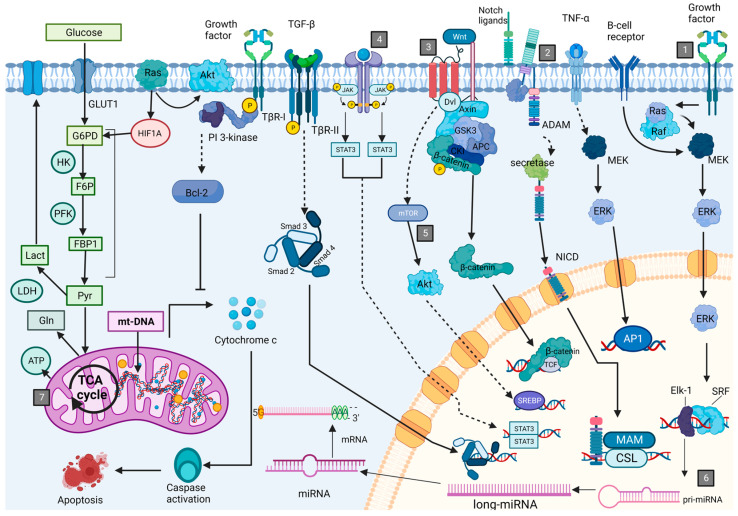
Cell signaling pathways involved in leukemia pathogenesis. Genomic and metabolic signaling pathways are dysregulated in BCP-ALL, affecting various aspects at different cellular levels: genetic, metabolic, and functional. Whether biomarkers are released in the peripheral blood (PB) depends on where the molecular machinery is affected. For example, the increase in activation of growth factor receptors (1) could lead to multiple downstream cascades, including Notch (2), Wnt (3), JAK-STAT (4), and PI3K/AKT/mTOR (5). This activation dysregulates control transcription factors, modifies the regulatory RNAs expression (6), and increases the mitochondrial metabolic activity (7) that triggers leukemogenesis development [51,52,53,54,55,56].

Those cellular processes are intrinsically regulated by signaling pathways, which mainly control transcription factors that triggers leukemogenesis development [12,24,25,42,43,44,45,46]. The most common pathways involved in leukemia pathogenesis are SCF/c-kit receptor, Notch, HOX, Wnt, EPO-induced, CXCL12-CXCR4, JAK-STAT, and PI3K/AKT/mTOR [35,36,47] (Figure 2). All the changes generated by leukemogenesis affect various aspects at different cellular levels: genetic, metabolic, and functional. Whether molecules are released in the PB depends on where the molecular machinery is affected. A DNA analysis may be used to identify which genes are altered because the disease and these changes may be useful to evaluate the prognosis of the disease and/or to predict changes in the RNA production of specific genes. An RNA quantification technique only allows for the analysis of gene expression at the messenger RNA (mRNA) level. Thus, researchers often employ transcriptomics, which provides information on gene activity in specific tissues. However, this technique does not offer information about for protein translation and post-translational modification. Proteomics fills this gap: It provides a perspective of biological processes at the protein level [48]. As we can notice, even though leukemia begins in BM, it produces many blood cells, proteins, and genomic material that enter the bloodstream. Therefore, analyzing these cells and/or molecules in PB is viable using various techniques, each with its benefits and drawbacks. [49,50]. 

### General Description of Main Type of Molecules with Potential as Biomarkers for BCP-ALL

A cancer biomarker is a biological molecule that suggests the presence of cancer, characterizes cancer, and can derive from cancer itself, or it indicates the response of the body to the existence of cancer [57]. A diagnostic biomarker is used to determine or confirm a type of cancer [57,58]. A prognostic biomarker is used to identify the likelihood of the outcome of the patients with cancer with or without treatment and on the disease recurrence or progression [57,58]. Circulating molecules with diagnostic and prognostic value may be located in PB and its components: plasma or serum, EV, and/or blood peripheral cells. In addition to the blood components, circulating biomarkers may be grouped according to their biochemical composition as proteins (enzymes, channels, matrix proteins, soluble proteins, surface proteins, intra or extracellular proteins, antibodies, receptors and ligands, etc.), RNAs (miRNA, mRNA, circRNA, and lncRNA, among others), cell-free DNA (cf-DNA: DNA, mtDNA), and metabolites (carbohydrates, fat acids, aminoacids, lipids, metals, and glycans, among others). 

## 3. Diagnostic Biomarkers for BCP-ALL

As mentioned in Section 1, the BCP-ALL diagnostic protocol is well established. Firstly, it is necessary to demonstrate ≥ 20% lymphoblasts in BM based on a BPM. Second, a hematopathological review is performed; it comprises a morphological assessment, and flow cytometric and genetic characterization. Once these studies are completed, a diagnosis according to the WHO classification can be made [12]. The BCP-ALL classification integrates morphology, immunophenotyping, and genetics/cytogenetics. There are no morphological features to distinguish between the BCP-ALL and TCP-ALL. Nevertheless, some lymphoblast characteristics are relevant: scant cytoplasm, size, and shape, wide relatively dispersed chromatin, and nuclear, and nucleolar peculiarities [59]. The immunophenotyping in BCP-ALL show some markers as almost always positive, namely CD19, cCD79a, cCD22, CD22, CD24, PAX5, and TdT; CD20, CD34, CD13, and CD33 expression is variable. Finally, among the genetic abnormalities BCR—ABL1, KMT2A-rearranged, ETV6-RUNX1, hyperdiploidy, hypodiploidy, IGH/IL3, TCF3-PBX1, BCR-ABL1-like, and iAMP21 can be mentioned [5]. 

The following circulating biomarkers have shown promise for either the diagnosis or prognosis of the illness.

### 3.1. Proteins Type BCP-ALL Biomarkers

Proteins are helpful biomarkers because they have multiple crucial functions, making them central in biological systems [48,60]. In an individual manner, tumor necrosis factor α (TNF-α) is among the most studied biomarkers for ALL. TNF-α is a cytokine that induces apoptosis by inhibiting the activity of caspases [61]. Ahmed et al. evaluated its levels in serum from adult patients with ALL, comparing patients who received chemotherapy to those who did not (Table 1 and Supplementary Table S1). Based on receiver operating characteristic (ROC) curve analysis, TNF-α showed good ALL diagnostic utility, an area under the ROC curve (AUC) of 0.94, a sensitivity of 91.7%, and a specificity of 100% [3]. These reports are consistent with the findings reported by Aref et al., who found an increase in TNF-α levels among patients newly diagnosed and in remission compared with controls [62]. Ahmed et al., was able to confirm this using an ROC curve, even though Aref’s cohort of patients had both BCP-ALL and BCP-CLL. These findings indicate that TNF-α can be a valuable tool in diagnosing ALL [3,62,63]. Table 1 summarizes the markers whose diagnostic values for ALL and/or BCP-ALL have been determined.

With the aim of highlighting a functional role of Smad 7, transforming growth factor β1 (TGF-β1), and miR-181a in clinical diagnosis and prognosis of pediatric-ALL, Nabhan M et al. evaluated miR-181 expression and Smad 7 and TGF-β1 protein levels in the serum of 60 children with ALL [16]. The authors reported lower TGF-β and higher levels of Smad7 expression in controls compared to patients. Smad 7 showed a correlation with hepatomegaly, splenomegaly, and lymphadenopathy [16]. The authors reported high AUC values, especially when they were combined (sensitivity = 100%; specificity = 93.3%; cutoff values: miR-181a ≤ 0.97, Smad7 ≥ 400.1 pg/mL, and TGF-β1 ≤ 0.25 ng/mL). Regarding cell proliferation, the TGF-β1 signaling pathway is regulated at different levels and can act as tumor suppressor or promotor depending on the cell type [16]. So, TGF-β1 regulates cell proliferation, differentiation, apoptosis, adhesion, and migration. To activate Smad proteins, a family of receptors is required to undergo phosphorylation and activation. These receptors belong to the kinase domain type I TGF-β, which play a crucial role in controlling the TGF-β signaling pathway. The results showed resistance to the growth inhibitory and apoptotic effects of the TGF-β1 signaling pathway due to elevated Smad 7 levels, thus meaning that the TGF-β1 signaling pathway showed to be involved in ALL pathogenesis [16]. 

The presence of autoantibodies VDAC1 (voltage-dependent anion-selective channel 1) (*p* < 0.05) and α-enolase (*p* < 0.05) has also been reported as overexpressed in the serum of patients with BCP-ALL [14]. Researchers have postulated that the presence of these proteins could be immunogenic at the early stages of the disease (although their mechanism remains unclear). Because of their functions, α-enolase and VDAC1 may be used as auxiliary tools for the diagnosis and immunological surveillance, treatment, and prognosis of pediatric BCP-ALL [14]. 

A valuable method for evaluating multiple proteins is “proteomics”, which evaluates the proteome. The proteome is a group of proteins expressed in a cell, characterized by their distinct localization, post-translational modifications, interactions, and turnover [48]. In leukemias, aberrant protein expression patterns can identify the pathogenesis of the disease, post-translational modifications, origin, and the differentiation of leukemic cells [7,48,60,78]. If successful, the study of one or a small number of proteins contributes to the understanding of the pathogenesis of disease; understanding the dynamics of the human proteome is crucial for developing biomarkers to be used as measurable indicators for disease severity and progression, patient stratification, and drug development. To distinguish between hematological malignancies with a proteome profile, researchers evaluated plasma from 107 patients older than 18 years with AML, ALL, or APLL, and 144 patients with lymphomas by using a proximity extension assay (PEA) [78]. PEA is a technology that translates protein information into actionable knowledge by linking protein-specific antibodies to DNA-encoded tags [79]. Regarding AL, the authors reported von Willebrand factor (vWF), SYND1, TNF-RSF6B, MPO, VIM, TNF-R1, IL-6, CTSD, and FURIN levels were remarkably higher compared to controls (fold change [FC] > 2; *p*-values < 1.0 × 10^−40^); meanwhile, ADAM-TS15 (FC = 0.43; P = 3.34 × 10^−40^) levels were lower. Moreover, the most differentiating proteins between ALL, AML, APL, and controls were TCL1A, CD27, and CD48 (Supplementary Table S1). They did not find significant protein expression differences between of AML and ALL, perhaps due to the small number of patients. Therefore, those proteins can distinguish between leukemia, healthy patients, and other hematological malignancies. The functions of altered proteins were associated with hemostasis, inflammation, and cell-to-matrix integration [78,80,81,82]. Speaking of hemostasis, vWF helps bind platelets to vascular injury sites after a proteolytic reaction regulated by FURIN [78,82]. These data are consistent with increased vWF and FURIN levels because thrombocytopenia and blood coagulation disturbances are crucial in acute leukemia. Similarly, the expression of the members of the TNF-R superfamily is involved in the inflammatory response and contributes to cell differentiation and survival [78,83].

In a study using high throughput technologies for proteins, Zhu et al. evaluated exosomal proteins in plasma from the blood samples of patients with BCP-ALL [68]. After isolating exosomes, they performed proteomic analysis by mass spectrometry and identified 342 differentially expressed proteins associated with Notch and the autophagy pathways, confirmed by Kyoto Encyclopedia of Genes and Genomes (KEGG) enrichment analysis. Because of this analysis and their potential role in BCP-ALL, the authors selected ATG3 and ADAM17 as candidate biomarkers for BCP-ALL progression. In the study, both proteins were overexpressed in patients with BCP-ALL, as verified by Western blotting. A high AUC value calculated for ADAM17 (AUC = 0.989, 95% confidence interval (CI): 0.960–1.018, *p* < 0.0001) and for ATG3 (AUC = 0.956, 95% CI: 0.885–1.026, *p* < 0.0001), indicating their potential as diagnostic biomarkers for BCP-ALL [68]. 

In another study, the authors evaluated the peripheral lymphocytes of 15 children diagnosed with ALL by using 2D electrophoresis, mass spectrometry, and identification in silico [4]. Among the 15 differentially expressed proteins, they could only identify eight. Glutathione S-transferase P (GSTP) (*p* < 0.01) and prohibitin (PHB) (*p* < 0.01) were upregulated. Peroxiredoxin 4 (PRDX4) (*p* < 0.01), the 60S acidic ribosomal protein P0 (*p* < 0.01), pyridoxine-5′ -phosphate oxidase (*p* < 0.01), triosephosphate isomerase 1 (*p* < 0.05), cytoplasmic actin (*p* < 0.01), and hypothetical protein FLJ26567 (*p* < 0.01), were downregulated; it is theorized that the expression levels of this last protein depends on the cell type. Although the authors were not able to speculate on the specific role of each protein in leukemogenesis, the whole panel of proteins demonstrated diagnostic value [4]. Similarly, in children with ALL, Shi et al. evaluated protein differentiation in serum blood samples. Mass spectrometry, bioinformatic analysis, and additional validation with immunoassays revealed that platelet factor 4 (PF4) (*p* = 1.54 × 10^−7^) and connective tissue activating peptide III (CTAP-III) (*p* = 7.19 × 10^−8^) were downregulated, and the fragments C3a-8137 (*p* = 5.35 × 10^−5^) and C3a-8937 (*p* = 5.13 × 10^−5^) were upregulated. Together, these proteins showed a sensitivity of 96% and a specificity of 98% for ALL diagnosis, implicating them as potential biomarkers for pediatric-ALL [7].

### 3.2. RNA Type BCP-ALL Biomarkers 

RNA can generate numerous proteins through protein-coding regions. Most genomic sequences can be transcribed into protein-coding RNA. In contrast, the non-coding parts are transcribed to produce non-coding RNAs (ncRNAs) [84,85]. Based on their size, location, and interacting partners, ncRNAs are classified into several types: transfer RNAs (tRNAs), ribosome RNAs (rRNAs), small nucleolar RNAs (snoRNAs), small nuclear RNAs (snRNAs), microRNAs (miRNAs), small interfering RNAs (siRNAs), long non-coding RNAs (lncRNAs), circular RNA (circRNAs), Piwi-interacting RNAs (piRNAs), and enhancer RNAs (eRNAs) [84].

It has been discovered that numerous microRNAs are essential in the initiation, progression, and metastasis of cancer [86]. miRNAs are stable in serum and display distinct expression patterns in healthy individuals and those with cancer. Hence, they are suitable biomarkers for cancer detection and prognosis [32]. Furthermore, one miRNA can modulate the expression of several genes, and there are many miRNA expression patterns in patients with ALL [87]. In a study carried out in Mexico, the authors evaluated the diagnostic usefulness of the circulating miRNA expression profile in plasma samples from patients with BCP-ALL. miR-511 showed the highest mean overexpression (FC: 159.5, *p* = 0.002), while miR-199a-3p was the most under-expressed (FC: −13.48, *p* < 0.001). ROC curves analysis provided good values for miR-511 (cut-off = 9.458, specificity 1, sensitivity 1, AUC = 1), miR-34a (cut-off = 7.179, specificity 1, sensitivity 0.92, AUC = 0.98), miR-222 (cut-off = −0.1325, AUC = 0.91), miR-26a (cut-off = 2.073, AUC = 0.91), miR-221 (cut-off = −0.1861, AUC = 0.92), and miR-223 (cut-off −4.309, specificity 1, sensitivity 0.89, AUC = 0.93). Moreover, pathway analysis revealed that mainly Wnt, MAPK, TGF-beta, p53, Jak-STAT, NOTCH, and B- and T-cell receptor signaling pathways are activated by the evaluated miRNAs (miR-511, miR-19b, miR-195, miR-565, miR-34a, miR-222, miR-363, miR-181a, miR-181c, miR-199a-3p, miR-340, miR-335, miR-99b, miR-221, miR-744, miR-223, miR-26a, miR-224, and miR-151-3p). These miRNAs have value as markers for diagnosis and to identify therapeutic targets for BCP-ALL [73].

CircRNAs are a type of non-coding RNA and represent a recent research hotspot in the field of RNA. circRNAs form covalently closed loop structures with neither 5′–3′ polarities nor polyadenylated tails and lengths between 100 to thousands of nucleotides [88,89]. circRNAs can modulate miRNA-target expression, acting like miRNA axes. Moreover, they can interact with RNA-binding proteins and regulate cellular processes [90].

**Figure 3:**
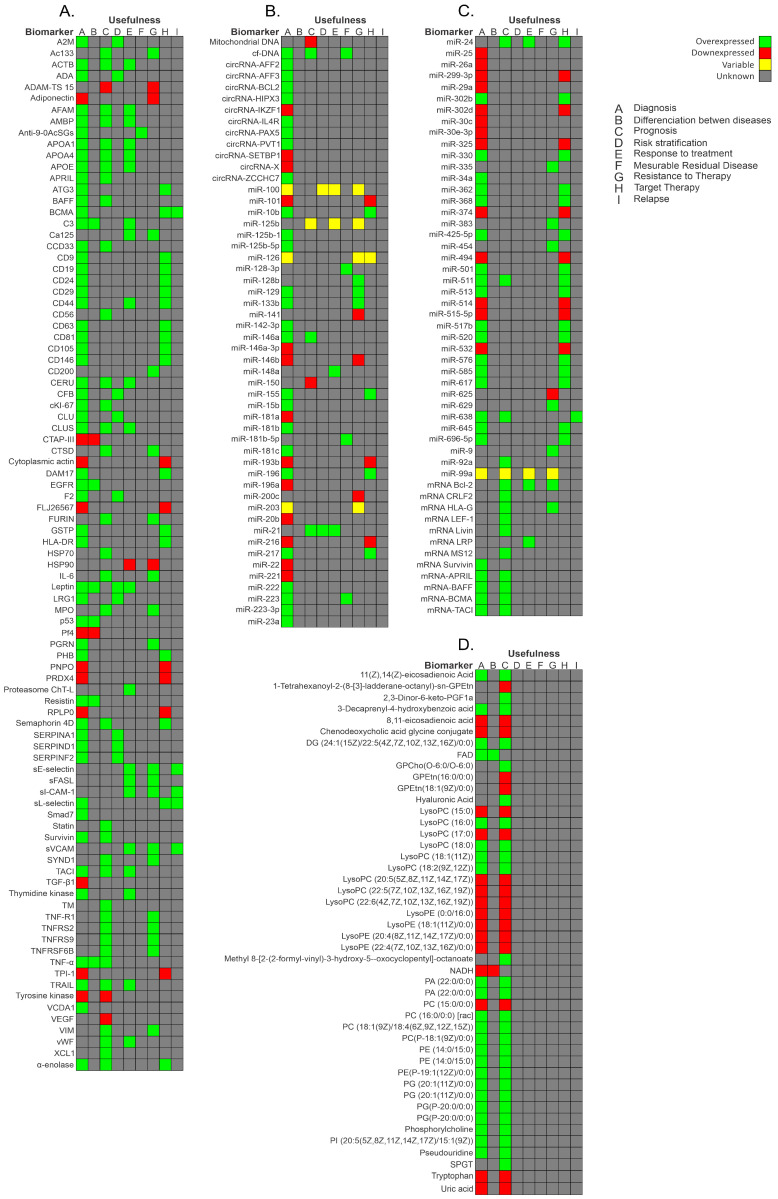
Biomarkers associated with BCP-ALL grouped according to their value in prognosis, risk stratification, treatment response, and evolution stages of the disease. The heat maps show the circulating biomarkers with positive association with BCP-ALL grouped as protein markers (**A**), nucleic acid molecules (**B**,**C**), and metabolites (**D**).

Circular RNA (circRNA) expression in PBMCs of pediatric patients with BCP-ALL has also been evaluated [90]. After the comparison of circRNA expression profiles in B- and T-cells, and monocyte populations, which was validated by reverse transcriptase quantitative polymerase chain reaction (RT-qPCR), four circRNAs were more highly expressed in healthy donors than in patients with BCP-ALL: circIL4R (*p* < 0.0001), circZCCH7 (*p* = 0.0307), and circX (intergenic) (*p* = 0.076). Furthermore, circPVT1 (*p* = 0.0002), circHIPK3 (*p* < 0.0001), circPAX5 (*p* < 0.0001), and circAFF3 (*p* = 0.0115) were overexpressed in patients with BCP-ALL. The expression of the target set of circRNAs according to BCP-ALL cytogenetic subtype showed circAFF2 was highly expressed in TCF3-PBX1 (*p* = 0.0252), BCP-ALL, and to a lesser extent in ETV6- RUNX1 BCP-ALL (*p* = 0.021). CircBCL2 (intronic) was upregulated in ETV6-RUNX1 fusions (*p* = 0.0166), circSETBP1 and circX (intergenic) were significantly reduced in MLL rearranged samples (*p* = 0.0274 and *p* = 0.0472, respectively), and circIKZF1 was lower in BCP-ABL and hyperdiploid leukemias than in the ETV6-RUNX1 subtype (*p* = 0.0154), in which the expression was conserved at levels comparable to B-cells. Overall, circRNAs have the potential to regulate specific cell functions, cell differentiation, maturation stages, and the growth of leukemic cells. As circRNA deregulation has been observed in patients with BCP-ALL, based on their cytogenetic subtype and previous cancers, circRNAs could potentially serve as markers for BCP-ALL. However, further research is necessary to determine the exact role that circRNAs play in leukemogenesis and their potential as markers for the disease [90,91]. Figure 3 shows a summary of the biomarkers associated with BCP-ALL grouped as protein markers (Figure 3A), nucleic acid molecules (Figure 3B,C), metabolites (Figure 3D), and according to the different stages of disease.

### 3.3. Metabolites Type BCP-ALL Biomarkers

Metabolites are the final products of gene expression and the direct outcome of enzymatic and protein activity. Metabolite profiles provide information about tumor microenvironments for ALL [30,92]. These metabolite profiles can be studied by metabolomics. “Metabolomics” involves the quantitative measurement of time of the metabolic responses of multicellular systems to pathophysiological stimuli or genetic modification [17]. Perturbation in the metabolism of patients at different disease stages could provide a unique metabolic signature to monitor treatment outcomes and disease progression [93]. Metabolism in cancer is a major research area in cancer biology. It examines how metabolic activities are changed in cancer cells compared to normal cells [30].

As shown in Table 1, Supplementary Table S1, and Figure 3D, researchers have reported metabolic differences in patients with ALL compared to healthy controls. Musharraf et al. [92] compared the metabolite profiles in the serum of 96 patients with ALL, AML, and APA, obtained by using the nuclear magnetic resonance spectroscopy technique. The authors reported high lactate levels and low levels of alanine, glutamine, histidine, lysine, valine, and proline. They theorized that patients adopt a secondary metabolic pathway to generate glucose because cancer cells require more glucose than can be provided by glycolysis. These findings are in contrast with those reported by Morad et al. [30], who evaluated the plasma of patients with ALL (*n* = 14), AML (*n* = 16), and breast cancer (*n* = 25). They reported high threonine, proline, glycine, alanine and lysine levels and low lactate levels in the ALL group. These changes could be due to tumor tissue competing for nitrogen compounds found in the amino acid structure [30,92]. Another group found that fatty acids were elevated as reservoirs, corresponding with an accumulation of carnitine, which plays a role in fatty acid metabolism [92]. Upon closer analysis, there seem to be notable variations in the approaches employed, which could explain the discrepancies in the outcomes. A crucial factor to consider is that the patient selection was comprised of diverse cancer types, like breast cancer and aplastic anemia, which may have influenced the results. Furthermore, though both studies utilized nuclear magnetic resonance spectroscopy, the validation techniques differed. One study used variable importance in projection values, while the other used principal component analysis. To gain a comprehensive understanding of the implications of these disparities, providing further context and specific examples would be highly beneficial. Overall, with a more thoughtful and informed examination, we can better appreciate the nuances of these findings. 

In a recent study focused on developing a novel photo-diagnostic strategy using fluorescence emission spectra, the authors used plasma or red blood cell extract from 45 patients with AL. They found four metabolites that were altered and correlated: nicotinamide adenine dinucleotide (NAD) + hydrogen (H) (NADH), FAD, tyrosine kinase, and tryptophan (*p* < 0.05). NADH and tryptophan were decreased in patients with ALL, and there was a connection between tyrosine kinase and tryptophane. However, FAD was increased in patients with AL. Thus, the AL diagnosis can be made based on the FAD, NADH, tyrosine kinase, and tryptophan levels, but the ratios can help to discern between AML and ALL [8]. 

## 4. Prognostic Biomarkers for BCP-ALL

Many disease- and patient-related factors affect the prognosis of BCP-ALL, including age, the white blood cell (WBC) count, and clinical features at diagnosis, cytogenetic abnormalities, pharmacodynamic and pharmacogenetic characteristics of normal patient cells, early response to therapy, and the measurement of MRD [17,18,19,20,21]. The primary use for prognostic identification is to define the treatment approach [12]. 

Notwithstanding the high rates of remission induction and survival achieved through the current treatment regimens, the response to therapy is still poor in a subset of patients. This fact necessitates a thorough comprehension of the survival signals and microenvironment that contribute to the establishment of a leukemic clone and its resistance to therapy. The implications for the dysregulation, which promotes survival and provides co-stimulatory signals, are still broad. The ability to manipulate such a powerful axis could increase or even grant total independence of environmentally regulated homeostatic control [94].

### 4.1. BCP-ALL Biomarkers for Prognosis and Risk Stratification

#### 4.1.1. Proteins

In addition to its use in diagnosis, TNF-α has prognostic significance. Higher TNF-α levels in patients with ALL (range: 50–476.4 pg/mL) than the control group (range: 20.53–190 pg/mL) (*p* ≤ 0.01) have been reported. Moreover, TNF- α levels in patients with ALL were lower after therapy (range: 44.47–384.47 pg/mL) compared to before therapy (range: 50.0–476.4 pg/mL). TNF-α levels also showed a significant correlation with Philadelphia-chromosome-positive ALL (*p* < 0.05) and the peripheral blood cell count in patients with ALL (*p* < 0.05) [3].

B-cell-activating factor (BAFF) and a proliferation-inducing ligand (APRIL) are members of the TNA superfamily. Sun et al. evaluated their expression in the plasma of untreated children with ALL and children in remission. APRIL levels were higher in untreated patients (range: 22.7–32.2 ng/mL) compared to normal controls (range: 15.8–18.0 ng/mL, *p* < 0.001) and the remission group (median 17.7 ng/mL, range: 16.1–19.8 ng/mL, *p* < 0.001). APRIL levels were also higher in patients with ALL and a WBC count of >50 × 10^9^/L (*p* = 0.002) and the risk group (28.3–47.8 ng/mL, *p* = 0.013). Moreover, the authors found a synergic relationship between BAFF and APRIL. They theorized that BAFF and APRIL form a heterodimer that regulates B- and T-cells in the pathogenesis of ALL [95]. Another group also evaluated APRIL levels in the serum of 48 patients with BCP-ALL. Patients with APRIL values higher than the median (5.51 ng/mL) had a higher OS (0.3, *p* = 0.01) and relapse-free survival (0.2, *p* = 0.03) than patients with lower concentration levels of the median values at 60 months [94]. Based on these findings, APRIL could be a valuable tool to evaluate ALL disease activity and prognosis. 

Among the tumor necrosis factor superfamily of ligands (TNFSF) and their receptors, (TNFRSF), TNFRSF9, TNFRF2, and fascin-1 have been evaluated in the plasma and leukocytes of 80 patients with AL. An increase in leukocyte TNFRSF9 level was observed in patients with ALL (range: 3.1–5.3, *p* < 0.05) and AML (range: 3.1–8, *p* < 0.05) compared to the control group (range: 3–5, *p* < 0.05), while plasma TNFRSF2 was elevated only in patients with ALL (range: 15.9–146.5, *p* < 0.05) compared to the control (range: 14–52.6) and AML patients (range: 11.5–249.2, *p* < 0.05) groups. Moreover, there was a significant increase in leukocyte TNFR2 levels in ALL (range: 16.1–74.7, *p* < 0.05) and AML (range: 13.2–235.5, *p* < 0.05) groups. Plasma fascin-1 was also increased in patients with AML (range: 4.2–21.1, *p* < 0.05). The median OS in patients with ALL was 14 weeks (95% CI: 5.7–18.3 weeks). TNFRSF9 levels increase as the patient’s immune response triggers leukemic cells to produce TNF, which reflects the severity of ALL. Consequently, TNFRSF9 and TNFRF2 may be good indicators of a poor prognosis for ALL, while fascin-1 can differentiate between AML and ALL [96]. 

Pediatric patients with ALL can show endothelial dysfunction before and after chemo/radiotherapy. This damage may be caused as a result of the disease itself, treatment, or other conditions like sepsis [97,98]. To assess markers of endothelial activation, Hatzipantelis et al. measured TM and vWF levels at diagnosis and induction (33 days of chemotherapy) in the whole blood samples of 53 children with ALL to correlate them with levels of acute phase reactants and risk factors. The authors reported a positive correlation between the leukocyte count and the levels of vWF (r = 0.333, *p* = 0.025) and TM (r = 0.583, *p* = 0.0001). Moreover, patients who had relapsed or died had higher leukocyte counts, and TM levels were linked in patients who relapsed or died (*p* < 0.05). Consequently, the elevation of these proteins in patients with ALL during the acute phase confirmed endothelial dysfunction. The correlation with the leukocyte count before treatment and the high levels of TM in children with unfavorable outcomes implies that TM and vWF levels might be prognostic markers in childhood ALL [98]. These results are consistent with Hagag et al., who established a relationship between the vWF and TM serum levels of 40 children with ALL and survival and remission rates after a 2-years follow-up. They found significantly higher TM and vWF levels in patients with complete remission (CR) (mean ± standard deviation (SD): 15.37 ± 2.64) than in those who had relapsed or died (mean ± SD: 22.59 ± 1.93, *p* < 0.001) [99]. During remission, the levels of TM and vWF were found to be elevated in both studies, indicating endothelial dysfunction in ALL. Hatzipantelis et al. also noted that P-selectin levels were higher after induction, suggesting that chemotherapy may contribute to this dysfunction. These findings indicate that these markers could potentially be important prognostic factors for childhood ALL [98,99].

Progranulin (PGRN), is a secreted protein that regulate cell cycle progression, cell motility, and tumorigenesis. Moreover, its participation in leukemogenesis has been suggested [100]. To determine the prognostic significance of PGRN, El-Ghammaz et al. evaluated its levels in the serum of 40 adult patients newly diagnosed with ALL. Patients with ALL had higher PGRN levels (range: 25–800 ng/mL) compared to controls (range: 10–35 ng/mL, *p* = 0.003). They found a clear correlation was shown between higher PGRN levels and a higher likelihood of relapse, as demonstrated by the cumulative incidence of relapse (CIR) (cut-off = 127.5 ng/mL, *p* = 0.008). Disease-free survival (DFS) was also affected by PGRN levels, as noted by the time from CR to relapse (cut-off = 127.5 ng/mL, *p* = 0.029). Thus, serum PGRN levels may be used to predict disease relapse [101].

Semaphorins are a large family of secreted, transmembrane, or glycosylphosphatidylinositol (GPI)-anchored proteins initially identified as axon guidance cues that signal through their receptors, namely neuropilins, and plexins [102]. Emerging evidence suggests they also function in a broad spectrum of pathophysiological conditions, including hematological malignancies [103]. Semaphorin 4D (Sema4D), also known as cluster of differentiation 100 (CD100), is encoded by the Sema4D gene in humans [104]. In vivo and in vitro studies on Sema4D have shown high levels of soluble Sema4D in pediatric patients with ALL. Patients with BCP-ALL had higher levels of Sema4D than controls (*p* < 0.01). OS has been analyzed in patients with AML [105]. Xue et al. correlated risk classification factors with the expression of Sema4D in the PBMCs, bone marrow mononuclear cells (BMMC), and serum from 22 pediatric patients newly diagnosed with ALL. Sema4D was highly expressed in patients with BCP-ALL, TCP-ALL, and AML compared to controls. There was a positive correlation between Sema4D levels in PBMCs and in serum (*p* = 0.0249), and between Sema4D levels in PBMCs, and the leukocyte count (*p* = 0.0002) and the peripheral blast number (*p* = 0.0036) [106]. As per the study conducted by Jian et al. [105], it was not observed that the difference in Sema4D expression varied according to the cell type. This is probably because the classification was BCP-ALL and non-BCP-ALL. However, additional in vitro experiments conducted on BALL-1 cells did reveal that a knockdown of Sema4D led to G0/G1 cell cycle arrest, increased apoptosis, and inhibited proliferation. On the other hand, Sema4D overexpression promoted cell division and proliferation while inhibiting apoptosis, and these effects were dependent on the cell origin. Moreover, Sema4D knockdown inhibited the phosphorylation of PI3K and AKT in the Jurkat and BALL-1 cell lines, implying that Sema4D activates the PI3K/AKT signaling pathway. These findings are supported by Sema4D expression in pediatric leukemia samples. Similarly, ERK phosphorylation was decreased in BALL-1 cells with Sema4D knockdown. That pattern was also observed in vivo and correlated with the level of expression. It is assumed that Sema4D plays a crucial role in leukemia development by regulating the PI3k/AKT and ERK signaling pathways and may be a biomarker for ALL prognosis [105,106]. 

Plasma circulating heat shock protein 70 kDa HSP70 (cHSP70) is believed to play a role in the anti-tumor immune responses, and its levels may reflect the severity of the disease condition [107]. To evaluate its association with disease prognosis, the cHSP70 levels were determined in the plasma of 40 patients with ALL and 96 patients with AML. The authors reported a significant correlation between cHSP70 plasma levels and the blast count in bone marrow (*p* = 0.04), β2 microglobulin (*p* = 0.043), the WBC count (*p* = 0.010), lactate dehydrogenase (LDH) (*p* = 0.001), and shorter survival (123 weeks vs. 350 weeks, cut-off = 55 ng/mL, *p* = 0.05), but only in patients younger than 60 years. The authors proposed that the prevalence of cHSP70 is derived from leukemic cell turnover. Nonetheless, it could also be secreted by lymphocytes or other organs and tissues in response to specific stresses and pathological conditions. Thus, HSP70 is an adverse independent prognostic indicator for survival in ALL and AML [107]. In another study, the authors demonstrated that stress causes the liberation of HSP70 from tumor cells and suggested it is reduced after cytokine treatment; hence, it could be beneficial for managing cancers like leukemia [108]. 

In a cohort-based population of Egyptian children with ALL and AML, Elgendi et al. evaluated the prognostic value of antigen 133 (AC133) [69]. The expression of AC133 was determined by flow cytometry in PB and BM blasts from 30 children with AML and 30 children with ALL, all of whom were CD34-positive and were followed up with for 12.5 ± 1.49 months. There was an association between patients with positive AC133 with ALL and those with no treatment response (*p* < 0.0001), early relapse (*p* < 0.0001), a shorter OS (4.7 ± 0.61, months vs. 27 ± 1.41 months, *p* < 0.001), and a shorter DFS (1.3 ± 0.43 vs. 26.2 ± 1.41 months, *p* < 0.001). They performed an evaluation with ROC analysis to predict cases of poor prognosis. Using a cut-off value of AC133 expression > 33.3% in patients with ALL, the sensitivity and specificity values were 100%. Nevertheless, they found no association with any known prognostic factor [69]. Because AC133 is expressed in CSC, which initiates ALL, AC133 can be considered an independent risk factor associated with poor response to chemotherapy and short survival [69,109].

For patients with BCP-ALL, the risk classification system uses factors that are simple to measure and, therefore, easily generalizable, including elements like age, the WBC count at the time of diagnosis, the immunophenotypic/cytogenetic/genetic subtype, and the presence of CNS infiltration. These factors allow for the division of patients into four risk groups: low, standard, high, and very high [12,44]. In a proteomic approximation, Braodaki et al. evaluated BM, PB plasma, and cell lysates from 45 pediatric patients diagnosed with ALL after classifying them into low or high risk according to their risk factors. Additional analyses in all tissues of origin, revealed the following proteins that could discriminate irrespectively of the sample between low- and high-risk patients were: ZA2G, ficolin-3 (FCN3), CFAB, apolipoprotein A4 (APOA4), apolipoprotein A1 (APOA1), AMBP, A1AT, and serum amyloid A protein (SAA) (*p* < 0.01). These proteins showed higher expression in high-risk patients. In contrast, ACTG, ceruloplasmin (CERU), apolipoprotein E (APOE), ANT3, clusterin (CLUS), ACTB, and VTB (*p* < 0.01) showed higher expression in low-risk patients. Of these proteins, FCN3 (*p* < 0.01), CFAB (*p* <0.01), CLUS (*p* <0.01), CERU (*p* < 0.01), and APOA1 (*p* < 0.01) were able to discern between normal and aberrant ALL karyotypes. Nevertheless, OS was significantly different for APOA1, CERU, SAA, and ACTB (*p* < 0.001). Lastly, Gene Ontology analysis proved that APOE, APOC2, APOA4, and APOA1 are involved in cholesterol regulation and the statin pathway, including cholesterol regulation and lipoprotein modeling. To summarize, CLUS, CERU, APOE, APO4, APOA1, S10A9, AMBP, ACTB, and AFAM might differentiate between low- and high-risk patients with ALL [110]. 

#### 4.1.2. BCP-ALL RNA Biomarkers

To evaluate clinically relevant molecules, Ibrahim et al. studied the expression of livin, a protein member of the inhibitor of an apoptosis protein family, in BMNCs and PBMCs from a cohort of 80 children with ALL. Livin expression levels were higher in patients who had better prognostic factors, such as ages 1–10 years (*p* = 0.03), patients with t (12, 21) (*p* = 0.00), patients with hyperdiploidy (*p* = 0.02), and patients with leukemic blast < 25% at day 7 of chemotherapy (*p* = 0.001). Additionally, patients with higher livin expression had a higher chance of achieving CR after treatment (82.4%, *p* = 0.05) and longer OS (8.45 vs. 7.23 months, *p* = 0.02) and DFS (7.61 vs. 4.64 months, *p* = 0.02) than those with low levels of livin expression. The authors presumed that the favorable prognosis associated with high livin expression could be because the cleaved form of livin is a proapoptotic regulator. Furthermore, its association with the response to treatment suggests that livin expression is linked to a faster apoptotic response of leukemic cells to apoptotic stimuli that originated from chemotherapy. Hence, livin mRNA is a potential prognostic factor associated with a good outcome [111]. 

Although the deregulated expression of the cytokine receptor-like factor 2 (CRLF2) has been studied more in children, Chiaretti et al. evaluated it in the peripheral blood or bone marrow blasts from 102 adult patients with BCP-ALL. They aimed to identify a correlation between the expression of CRLF2 and the clinical and biological characteristics and outcomes of patients without genetic aberrations. Hence, they excluded patients with the fusion genes BCR/ABL1, ETV6/RUNX1, E2A/PXB1, MLL/AFF1, MLL1/ENL, SIL/TAL1, SET/NUP214, and NUP98/RAP1GDS1. They reported that CRLF2 expression levels were reasonably associated with hyperleukocytosis (*p* = 0.0002) and thrombocytopenia (*p* = 0.034) at diagnosis, along with a reduced OS (at 36 months: 95% CI: 8.5–50 and 76.5%, 95% CI: 52.1–89.6%, *p* = 0.0038) and DFS (20%, 95% CI: 3.1–47.5 vs. 71.1%, 95% CI: 43.8–86.9, *p* = 0.015) compared to patients with a low expression value. They also reported an association between mutations in the JAK/STAT pathway mutation in 65% of CRLF2 overexpressing cases (*p* < 0.0001). Conversely, the P2RY8/CRLF2 transcript was positive in two patients with CRLF2 overexpression [112]. Because deregulated CRLF2 expression has been described in patients with BCP-ALL lacking the above-mentioned genetic aberrations, the results suggest that only a subclone of leukemic cells carries this aberration sufficiently to drive CRLF2 overexpression. Taken together, CRLF2 mRNA is a potentially unfavorable prognostic marker of long-term outcomes in adult patients with BCP-ALL [112,113].

One oncogene implicated in leukemogenesis is the Musashi-2 gene (MSI2), an RNA-binding protein that induces or represses translation [104]. MS12, has been associated with chronic-lymphoblastic-leukemia-cell survival and proliferation, and poor prognosis in patients with CLL [114]. To validate the prognostic significance of MSI2 in ALL, Aly et al. evaluated MSI2 gene expression in the blood samples of 140 adult patients with BCP-ALL. The authors excluded patients who were BRC-ABL1-positive due to their specific therapy. MSI2 expression levels were higher in patients with BCP-ALL compared to controls (range 0.8–7.5 vs. 0.01–0.5, *p* = 0.001). Additionally, OS was shorter in patients with high MSI2 expression compared to patients with lower MSI2 expression (30-year OS, *p* = 0.018). Similarly, DFS was shorter in patients with high MSI2 expression than in patients with low MSI2 expression (28.2%, *p* = 0.008). Thus, MSI2 mRNA expression is an independent biomarker for an unfavorable prognosis in adults with BCP-ALL [27].

- miRNAs

A myriad of miRNAs map to regions of chromosomes associated with leukemia. They can act as tumor suppressors as well as oncogenes, called oncomiRs. One of the most evaluated oncomiRs in children and adults is miR-146a. Shahid et al. evaluated the plasma of 66 children and adults diagnosed with ALL to evaluate miR-146a as a potential biomarker for ALL [9]. It was overexpressed in patients compared to controls (FC ALL: 33.46 ± 15.84; FC BCP-ALL: 34.74 ± 16.19; and FC controls: 1.36 ± 1.03; *p* < 0.0001). ROC curve analysis (AUC = 1, 95% CI: 0.96–1.00) confirmed the utility of miR-146a for diagnosis. Of note, these values were reduced after chemotherapy (a decrease from 32.95 ± 15.74 to 0.8865 ± 0.754 in male patients with ALL, *p* < 0.0001; a decrease from 35.55 ± 16.68 to 1.6 ± 1.196 for female patients with ALL, *p* < 0.0001), further indicating the prognostic significance [9]. In contrast, Apiknar et al. reported that in the plasma of adult patients with AL, the fold levels of miR-146 (range: AL 0.01–704.38 × 10^3^ for AL and 26.39–35.38 f, *p* = 0.001) increased after 1 month of induction. However, expression data after treatment was not shown [115]. There were consistent results in PBMCs from pediatric patients with ALL; miR-146a was overexpressed in patients with ALL compared to pre-BCP-ALL and TCP-ALL (range: 91–222.55, 14.3–63, and 117.6–174.6, respectively; *p* < 0.0001). In another study, the authors validated the diagnostic utility of miR-146 by ROC curve analysis (AUC = 1, 95% CI 0.956–1, cut-off = 3.727, *p* < 0.0001) and reported the prognostic relevance of the WBC count (CC = −0.421, *p* = 0.001) and the lymphocyte count (CC= −0.337, *p* = 0.01) to miR-146 levels [15]. On the other hand, its prediction targets were also investigated, finding TRAF6, IRAK1, and IRAK 2 [9]. In addition, the PI3/AKT, Apop, PTEN, and NF-KB signaling pathways are potentially involved in children with ALL and a high WBC count [116]. Thus, these factors play a potential role in improving therapy development. Overall, miR-146a may be a biomarker for diagnosis, and prognosis, indicating poor OS for patients and good treatment outcomes in childhood and adult ALL [9,116].

MiR-100 is another oncomiR and is located on human chromosome 11 (HSA11) [117]. It has been reported to be overexpressed in children with ALL and validated with ROC calculation (AUC = 0.87, 95% CI: 0.779–0.934, sensitivity 82.72%, specificity 100%, cutoff 3.029, *p* = 0.0001), showing its utility as a diagnostic biomarker [15]. Schotte et al. evaluated the efficacy of several chemotherapeutics in vitro and reported a role for miR-100 in treatment resistance. They found an upregulation of miR-100 in the resistance to vincristine (FC > 14, *p* = 0.002) and daunorubicin (FC > 19, *p* = 0.041) [118]. In their research, Ramani et al. found that miR-100 was downregulated in ALL while also conducting an analysis of the mRNA and biological pathways affected by various miRNAs. They discovered that the expression of the GEMIN7, ABTB1, KRAS, IKBKE, and NEUROD1 genes were upregulated in patients with high WBC, while 9orf78, CEBPG, AKAP8, LAMTOR3, POLE3, TMEM87A, SURF4, RHEB, CSNK1D, RUNX3, ICOS, NOTCH1, RELA, TMEM9B, WDR82, ZBED3, ZFP91, HSPA14, CHD1, TAB2, MAFB, MKRN1M, PPM1D, CHUK, ATP2A2, NUPL1, PPP2R2A, YY1, RB1CC1, TCF7L2, SIRT1, and TNRC6A were downregulated. Based on their thorough analysis, they concluded that miR-100 regulates the FKBP51 and IFG1R/mTOR signaling pathways, ultimately suppressing proliferation and promoting apoptosis [116]. When examining the research conducted by Ramani et al., it is crucial to take into account that their results were obtained through the analysis of miRNA and gene expression data utilizing the leave-one-out cross-validation procedure as the prediction and validation model. This method was used to identify miRNAs and genes with predictive power after analysis in various published data and package software. It is important to note that this validation model tends to have a high variance, which is why the outcomes they obtained differ from the research carried out by Swellan et al. and Schotte et al. [15,116,118] 

In 75 children with BCP-ALL, researchers evaluated miR-21 from BMCP and PMCP collected at diagnosis and then a follow-up. There was an upregulation of miR-21 in patients with BCP-ALL compared to controls (9.62 ± 3.23 and 2.56 ± 0.83, respectively, *p* < 0.001), as well as an association between high miR-21 high levels and age < 2 or > 10 years (*p* = 0.00), a lower platelet count (*p* = 0.003), central nervous system (CNS) infiltration (*p* = 0.001), and the risk of drug resistance (MRD) at 14 and 28 days (*p* = 0.012 and 0.025, respectively). These results correspond with those reported by El-maadawy et al., i.e., miR-21 was overexpressed in the PBMCs of 43 pediatric patients with ALL that did not respond to treatment (*p* < 0.05), based on the total XIIB regimen. Moreover, a ROC curve analysis showed that miR-21 could distinguish between healthy children and patients with ALL (cut-off = 1.385, AUC = 0.565, 95% CI: 0.44–0.691, sensitivity 44%, specificity 55%, *p* = not significant) [74]. To summarize, miR-21 is a biomarker associated with poor prognosis and resistance to treatment and has no diagnostic usefulness. 

#### 4.1.3. Mitochondrial DNA

Mitochondria are the powerhouse of cells; the energy they produce allows cells to function. In solid tumors, mitochondria work even harder in an attempt to satisfy the abnormally high energy demands of malignant cells. This dysfunction can lead to mutations in mtDNA. Mitochondrial DNA (mtDNA) can also be found in circulation. mtDNA is usually found inside of the mitochondria, and it corresponds to a circular genome of 16,569 bp. It encodes ribosomal RNAs (rRNAs), transference RNAs (tRNAs), and 13 polypeptide subunits essential for oxidative phosphorylation (OXPHOS). mtDNA is a valuable biomarker to assess oxidative damage because it lacks DNA repair systems and the protection of genetic material by histone proteins. Nevertheless, dysfunction in mitochondrial metabolism is reported in human cancers. mtDNA has been found to decrease and increase in cancer tissues, and it correlates with chemotherapy resistance and cancer progression. In several studies, these mtDNA changes were observed at different stages of leukemia [119,120,121]. Researchers extracted total cellular DNA from PBMC and BCMC from 51 Indian children with ALL; their age-matched siblings and healthy individuals without a history of inflammatory disorders served as controls. They reported an increase in the mtDNA copies number in bone marrow samples from patients with ALL compared to controls (median: 765.4 DNA copy number vs. 176.7 DNA copy number, *p* < 0.0001). The mtDNA copy number was also higher in patients compared to their siblings (median: 211.6 DNA copy number, *p* = 0.0007). They reported a correlation only between an increased mtDNA copy number in patients with ALL ≥ 10 years (median: 1105) and not in patients < 10 years old (median: 528.6 DNA copy number) (*p* = 0.0221). On the other hand, mitochondrial deletion ratios were higher in patients with ALL compared to controls (0.495 vs. 0.1386, *p* = 0.0018). The researchers compared EFS between patients according to the median mitochondrial DNA copy number (765.4 DNA copy number, *p* = 0.04), revealing that EFA was increased in patients with a reduced mtDNA copy number (*p* = 0.04). Hence, mtDNA copy is a potential biomarker for predicting survival in ALL patients and poor prognosis [119].

#### 4.1.4. Metabolites

Bai et al. generated metabolomic profiles of children with ALL: some had not yet started treatment, and others were in remission. Based on a principal component analysis (PCA) score, they identified 30 to discern between healthy controls and patients with ALL. Phosphorylcholine; LysoPC(16:0); LysoPC(18:0); PC(P-18:1(9Z)/0:0); 3-Decaprenyl-4-hydroxybenzoic acid; DG(24:1(15Z)/22:5(4Z, 7Z,10Z,13Z,16Z)/0:0); LysoPC(18:2(9Z,12Z)); PE(P-19:1(12Z)/0:0); LysoPC(18:1(11Z)); PC(16:0/0:0)[rac]; PC(18:1(9Z)/18:4(6Z,9Z, 12Z,15Z)); PI(20:5(5Z,8Z,11Z,14Z,17Z)/15:1(9Z)); LysoPE(0:0/16:0); LysoPE(18:1(11Z)/0:0); LysoPc(15:0); LysoPC(20:5(5Z,8Z,11Z,14Z,17Z)); LysoPC(22:6(4Z,7Z,10Z,13Z,16Z,19Z)); LysoPC(17:0); LysoPC(22:5(7Z, 10Z,13Z,16Z,19Z)); and 8,11-eicosadienoic acid showed a higher intensity in patients without treatment than those in remission. On the contrary, PC(15:0/251 0:0); 11(Z),14(Z)-eicosadienoic acid; PA(22:0/0:0); PE(14:0/15:0); PG(20:1(11Z)/0:0); PG(P-20:0/0:0); uric acid; chenodeoxycholic acid glycine conjugate; LysoPE(22:4(7Z, 10Z,13Z,16Z)/0:0); and LysoPE(20:4(8Z,11Z,14Z,17Z)/0:0) (P < 0.05) showed a lower intensity in patients without treatment compared to those who were in remission. From a general view, they reported that the metabolic pathways affected were glycerophospholipid metabolism, GPI-anchor biosynthesis, primary bile acid biosynthesis, and purine metabolism. Hence, those metabolites could help to differentiate between patients with ALL and healthy children as well as potential prognosis markers due to their PCA score according to the patient treatment phase [17]. 

In another study with a similar metabolomic methodology carried out in a group of 21 children with ALL, Bannur et al. identified seven potential differently expressed metabolites in patients with risk of disease onset or relapse (FC ≥ 2, *p* < 0.01): 2,3-dinor-6-keto-PGF1a, GPEtn(16:0/0:0), GPCho(O-6:0/O-6:0), GPEtn(18:1(9Z)/0:0), methyl 8-[2-(2-formyl-vinyl)-3-hydroxy-5-oxocyclopentyl]-octanoate, 1-tetrahexanoyl-2-(8-[3]-ladderane-octanyl)-sn-GPEtn, and GPCho(O-2:0/O-1:0) [93].

### 4.2. Response to Treatment

Early response to treatment can be measured by methods that are not related to MRD, like widely available peripheral blast counts by light microscopy on day 8 of chemotherapy [122]. A poor prognostic sign is a poor PB response to 1 week of systemic steroid (and a single dose of intrathecal methotrexate) before multiagent induction chemotherapy. Other means of assessing the rapidity of the response to initial therapy, such as the PB response after steroid prophase, the PB response early in induction therapy, and the BM response at early time points within induction, have been shown to have prognostic significance [19,26].

#### 4.2.1. Induction

Leucine-rich alpha-2-glycoprotein 1 (LRG1), CLUS, thrombin (F2), heparin cofactor II (SERPIND1), alpha-2-macroglobulin (A2M), alpha-2-antiplasmin (SERPINF2), alpha-1 antitrypsin (SERPINA1), complement factor B (CFB), and complement C3 (C3) proteins have been reported as overexpressed in patients with newly diagnosed BCP-ALL compared with controls (FC > 2, *p* < 0.05). However, after comparing 96 patients who underwent induction treatment with the protocol of the Brazilian Group for Treatment of Childhood Leukemia (GBTLILLA-2009) [123] with healthy children, there were no significant differences observed (*p* < 0.05). There was evidence of protein–protein interactions and signal transduction, so it is presumed that they could be used as biomarkers to evaluate the pre-diagnosis of BCP-ALL. The authors found that nine proteins could serve as biomarkers to indicate treatment response, as all patients achieved complete remission. However, other factors, like assigning risk factors and determining the treatment regimen, must also be considered [124].

In the serum of 48 children with ALL, the levels of FAS and its ligand (FasL) were measured when the patient achieved remission during induction. The Fas levels were higher in patients with hepatomegaly (265.9 ± 53.2 vs. 236.1 ± 33.2 pg/mL, *p* = 0.026) and splenomegaly (262.9 ± 51.7 vs. 237.1 ± 34.8 pg/mL, *p* = 0.047). Additionally, OS was longer (394 ± 69.9 days vs. 254 ± 24 days, *p* < 0.018) and the time to CR was longer (380 ± 65 days vs. 346 ± 26 days, *p* < 0.013), in FasL-positive patients but not FasL-negative patients. These correlations confer prognostic relevance to FasL and can be taken as a favorable response to therapy [125]. 

Ki67 is a nuclear antigen that is an excellent marker of active cell proliferation in the normal and tumor cell populations. Researchers evaluated circulating Ki-67 (cKi-67) activity in the plasma of 27 ALL patients, and K562 and RAJI in vitro cells, to investigate its prognostic relevance. In cell lines, Ki-67 was downregulated during growth arrest. Moreover, the authors collected plasma samples from patients newly diagnosed with ALL at the beginning of induction, showing that cKi-67 levels were higher in newly diagnosed patients (range: 0–4574 U/100 μL, *p* < 0.001). In older patients with ALL (> 70 years), cKi-67 was also higher (*p* = 0.05). They determined a Ki-67 cut-off of 1500 units/100 μL of plasma, and patients of the upper quartile showed a shorter OS (208 weeks, *p* = 0.05) [126]. Volm et al. reported similar findings. They measured statin and Ki-67 in blast cells of PB or BM of children with ALL at diagnosis and after starting treatment. They detected a favorable survival (based on Kaplan–Meier analysis) for patients after starting chemotherapy (OS was higher in patients with a Ki-67 level > 5%, *p* = 0.07) [46]. In spite of the usefulness of cKi-67 as a diagnosis marker, the results of these investigations showed *p*-values close to statistical significance when the utility of cKi-67 as a poor prognosis marker or for the monitoring the efficacy of the therapy were evaluated; therefore, additional studies are needed to validate the clinical significance of this protein at those levels [46,126].

In the pediatric population, researchers evaluated cancer antigen 125 (Ca-125), a type of tumor marker. Ca-125 was evaluated in 30 children with ALL, 14 with non-lymphoblastic leukemia (ANLL), 31 children with non-Burkitt’s NHL (NBNHL), 11 children with Birkitt´s lymphoma (BL), and 17 children with Hodgkin´s lymphoma (HL) at the time of diagnosis and after 4 weeks of chemotherapy when the children had achieved CR. The most remarkable finding was the very high Ca-125 level (3099 IU/mL) in the single patient with ALL and pleural and pericardial effusion; this level is much higher in the AL group compared to standard upper limit (> 35 IU/mL). The author also found that Ca-125 levels were significantly higher in the AL group compared with the control group (*p* = 0.000). Moreover, in patients with leukemia, Ca-125 levels significantly decreased when they entered remission (*p* = 0.001). Overall, Ca-125 levels can be used to determine whether a patient is in remission if it was elevated at diagnosis. Moreover, it may indicate serosal infiltration despite no clinical findings [127]. 

Heat shock protein 90 kDa (HSP90) is a chaperone that promotes the maturation, structural maintenance, and proper regulation of specific target proteins involved, for instance, in cell cycle control and signal transduction [104] among other processes and has a remarkable participation in hematological malignancies, including leukemia [128]. Researchers measured HSP90 protein levels in a pediatric group of patients with ALL before treatment and after 33 days of the Berlin–Frankfurt–Munster (BFM) protocol [129,130]. There was a decrease in levels of HSP90 after induction (range at diagnosis 1.22–23.85 ng/mL: range at day 33rd 1.07–52.51 ng/mL); these findings suggest that cancer cells are sensitive to steroids and that HSP90 is related to a poor response to steroids in children with ALL [130].

In addition to proteins, miRNAs have been evaluated and have shown usefulness in monitoring the therapeutic response in leukemia. As an example, miR-125b expression levels were evaluated in a cohort of 120 pediatric patients, using PBMC and BMMC samples at diagnosis and at day 28 of induction according to the BFM guidelines [131]. The authors reported a low miR-125b expression at diagnosis in BM samples compared to healthy controls (mean ± SD 0.97 ± 0.34 vs. 2.40 ± 0.83, *p* < 0.001). ROC analysis revealed a cut-off ≤ 1.51, an AUC of 0.997, a sensitivity of 98.33% and, specificity of 96.67%. Moreover, the reduced miR-125b expression was associated with high-risk groups, such as age (*p* ≤ 0.05), a high bone marrow blast count at diagnosis and at day 15 of induction (*p* < 0.001), and a low hemoglobin concentration (*p* < 0.001). The evaluation of PBMCs on day 28 after induction revealed that miR-125 was significantly increased in patients with ALL (FC = upregulated = 1.7, *p* < 0.001). Regarding the outcome, BM samples helped reveal that patients with ALL who expressed miR-125b at a low level had shorter leukemia-free survival (at diagnosis: r = 0.299, *p* = 0.001; at day 28: r = 0.399, *p* = 0.000) and OS (at diagnosis: r = 0.264, *p* = 0.004; at day 28: r= 0.316, *p* = 0.000) [76]. These findings are not consistent with those reported by Schotte et al., who evaluated the role of miR-125b in chemotherapy resistance in the PBMCs or BMMCs of 81 children with ALL. They found the overexpression of miR-125b and resistance to vincristine (FC = −25, *p* = 0.001) and daunorubicin (FC = 20 overexpressed, *p* = 0.033) were correlated [118]. Nevertheless, this result can be linked to the study by Swellam et al., who reported that miR-125b-1 is overexpressed in children with ALL compared with controls (median level 66.67 vs. 2.04, *p* < 0.001). Still, patients with chromosomal translocation showed a higher median value. Moreover, they validated the diagnostic capacity of miR-125b-1 with ROC curve analysis (AUC = 0.858, 95% CI: 0.715–0.847, sensitivity 83.72%, specificity 100%, cutoff 3.209 copies, *p* < 0.0001). Hence, miR-125 is a potential diagnostic and poor prognostic biomarker for ALL. The expression of miR-125b varies depending on the tissue it is taken from. El-Khazragy et al. reported that it was downregulated in BM, but further analysis showed that it was in fact overexpressed in PB after induction. Therefore, miR-125b can be used as a biomarker to monitor treatment response, and it may indicate poor prognosis. [72,76,118].

In a similar approximation and with the aim to evaluate changes in the miRNAs profile during treatment, Nemes et al. evaluated miRNAs expression in the cell lines culture and the PMNCs or BMNCs of 51 children with ALL (at diagnosis and at days 15 and 33 of treatment under the ALL IC-BFM 2002 protocol, including following up with patients at 28.1 months). The authors reported the expression dysregulated levels of miR-16, miR-21, miR−24, miR−29b, miR−128b, miR−1 42-3p, miR−155, miR−223, and miR-128 in patients with ALL. By comparing the results of human cell lines, they found that miR-128 was the most overexpressed in vivo and in vitro. miR-128b was highly overexpressed at diagnosis in patients with good prognostic factors, as confirmed by an ROC curve analysis with an FC cut-off of 80. Lower levels of miR-128b (FC < 80) were associated with a poor prognosis and poor response to prednisolone on day 8; at relapse, they were significantly higher compared to values measured at initial diagnosis [77]. Overall, miR-128b is a good prognostic marker and could help to predict the prednisolone response, to follow up the response to therapy, and to detect early relapse.

#### 4.2.2. Consolidation

Consolidation therapy, also called “post-remission therapy,” is used after cancer is in remission following induction therapy. The goal of consolidation therapy is to lower the number of residual leukemic cells or to eliminate them completely to prevent relapse.

In a pediatric population of 112 children with ALL, Krawczuk-Rybak et al. evaluated the proteasomal activity of peripheral blood plasma at diagnosis and during treatment, following the ALL-IC 2002 protocol (based on the BFM protocol) [131]. The authors reported that patients with ALL, levels of chymotrypsin-like (ChT-L) were higher at diagnosis than in healthy children (mean ± SD: 6.3 ± 12.0 vs. 1.3 ± 0.5, *p* = 0.001). Moreover, ChT-L activity was higher in the patients who presented a high baseline leukocytosis (>50.0 × 10^9^/L) (*p* < 0.001), and it also showed a correlation with serum LDH activity (r = 0.273, *p* = 0.012). However, ChT-L activity was significantly higher in patients with LDH > 2000 IU/mL (median 4.8 U/mg) compared to patients with LDH activity in the range of 800–2000 IU/mL (median 2.3 UI/mL, *p* = 0.003) and < 800 IU/mL (median: 2.6 U/mg, *p* = 0.001). Otherwise, in ALL patients, ChT-L levels decreased after induction at day 33 compared to the day before treatment (range: 1.3–2.6 U/mg vs. 1.8–5.5 U/mg, *p* < 0.006; *p* = 0.006), and before the intensive treatment, ChT-L levels lowered to normal range (range: 0.6–1.8 U/mg). They found that the ChT-L correlation with the WBC count and LDH was higher at diagnosis; hence, ChT-L may be proportional to the tumor mass. Therefore, after completing induction therapy, the ChT-L levels did normalize in patients with ALL. This phenomenon may indicate the insufficient eradication of blast cells or the result of intensive blast lysis because of therapy. Thus, ChT-L is a potential biomarker in children with ALL to monitor the response to chemotherapy [132].

In a cohort of 33 patients with ALL, researchers evaluated the levels of X-C motif chemokine ligand 1 (XCL1) in the serum of peripheral blood at diagnosis, at the end of remission induction, and at the end of consolidation. Serum XCL1 Levels showed a gradual reduction over time (range: 0–313.5, 0–128.9, and 0–277.5 pg/mL, respectively). In addition, higher XCL1 levels at diagnosis were correlated with lower age and higher survival. An XCL1 level > 50 pg/mL at the end of remission-induction chemotherapy correlated with a high survival rate. The researchers reported a more prolonged survival time (median: 280 days) in the group of patients whose XCL1 levels decreased progressively during treatment. According to the authors, this relationship with survival at diagnosis and during treatment can be considered essential prognostic factors, explained by a progressive reduction in the leukemic burden in response to treatment. However, a larger sample and additional studies to evaluate XCL1 secretion are needed. Nevertheless, XCL1 is a potential biomarker associated with good outcomes in patients with ALL, making it worthwhile to assess response to treatments [21]. 

#### 4.2.3. Maintenance

Adipokines, contribute to the development and progression of solid tumors, which are surrounded by adipose tissue. There is accumulating evidence that they might play a similar role in the context of leukemia in the local BMM but also systemically via endocrine routes of interaction [133]. Several authors have studied adipocytokines in patients with ALL and AL [134,135]. Moshovi et al. evaluated the potential of adiponectin, leptin, and resistin in the plasma of nine children newly diagnosed with ALL. Plasma samples were taken at six time points per patient according to the HOPDA 97 protocol: (1) at diagnosis, (2) at the end of induction, (3) before the third cycle of the maintenance phase of chemotherapy, (4) before the sixth cycle of the maintenance phase of chemotherapy, (5) before the eighth cycle of the maintenance phase of chemotherapy, and (6) before the 10th cycle of the maintenance phase of chemotherapy. The authors reported that at diagnosis, when comparing the levels in patients to those of the controls, adiponectin was low (12.6 ± 2.3 vs. 23.2 ± 2.4 mg/mL, *p* < 0.0001 mg/mL), while leptin (27.4 ± 4.2 vs. 17.8 ± 3.4 ng/mL, *p* < 0.001 ng/mL) and resistin (5.2 ± 1.2 vs. 3.2 ± 1.1 ng/mL, *p* < 0.001) were high. Considering these values as references, before the end of the maintenance phase, adiponectin levels increased (16.6 ± 2.9 mg/mL, *p* = 0.024); meanwhile, leptin (17.1 ± 3.9 ng/mL, *p* = 0.018) and resistin (3.4 ± 0.9 ng/mL, *p* = 0.020) fluctuated and stabilized at lower levels. The fluctuation in adiponectin levels suggests that the hormone provides a compensatory mechanism for catabolic–anabolic imbalance in children with cachexia. Resistin had a similar behavior to leptin, but in a less remarkable way [134]. Aref et al. reported similar findings after evaluating serum levels of adiponectin and leptin in 80 patients with AL [135]. They further classified the patients according to the cytogenetic into favorable, intermediate, and unfavorable. They documented a correlation between the highest levels of leptin and the unfavorable group of patients (range: 5.4–22.5, *p* = 0.00), and between higher levels of adiponectin and the favorable group of patients (range: 7.2–10.5, *p* = 0.00). Moreover, leptin levels were higher in patients with ALL than in controls (range: 5.4–22.5 vs. 3.0–15.4, *p* = 0.00), and adiponectin levels were lower in patients with ALL and AML (range 9.8–15.5 in ALL, and 0.7–9.6 in AML, and 12.9–22.0 in controls, *p* = 0.01). Finally, leptin was positively correlated with the percentage of bone marrow blasts (*p* < 0.001), the WBC count (*p* > 0.01), and LDH (*p* < 0.01) in patients with ALL. Overall, these adipocytokines may be markers of responsiveness to chemotherapy in ALL, whereas adiponectin and leptin are potential prognostic biomarkers associated with poor outcomes in patients with ALL [134,135].

#### 4.2.4. Relapse

The relapse of leukemia can be categorized based on different factors. For example, isolated BM relapse refers to ≥ 25% blasts in the BM after complete CR with no other affected areas. On the other hand, combined relapse is defined by ≥ 5% lymphoblast reappearance in the BM with one or more extramedullary sites affected; nevertheless, a biopsy may be necessary [136]. Late and early relapses are also distinguished based on the time of recurrence. An early relapse is when the disease reoccurs less than 18 months from the initial diagnosis for isolated or combined BM relapse. A late relapse is when the disease reoccurs after 36 months from the initial diagnosis for isolated or combined BM relapse or extramedullary relapse [11].

Several studies endorse the use of proteins as a diagnostic and prognosis biomarker in multiple lineages of leukemia. O´Neil et al. [137] evaluated serum TK1 levels in peripheral blood from 33 patients with ALL at different stages of the disease, namely, pre-treatment, relapse, and remission. They reported higher TK1 levels in patients with ALL (*p* < 0.05), specifically in patients who relapsed (*p* < 0.05). Furthermore, the authors collected serial samples for 360 days to monitor TK1 activity. Serum TK1 activity was high at the beginning of treatment and then gradually decreased and stayed low in patients who went into remission [137,138]. These results are similar to those reported by López-Martínez et al., who evaluated serum TK1 levels in 125 children with AL. Of the 125 patients, 90 were diagnosed with BCP-ALL and showed a lower TK1 levels than patients with TCP-ALL or AML (range: 35–118 IU/L, 288–2108 IU/L, and 88–750 IU/L, respectively, *p* = 0.001). Nevertheless, most patients with BCP-ALL group and high TK1 levels had high-risk factors (age and leukocytes) [139], hence the substantial increase in TK1 levels in patients with AL, along with its correlation to leukemia subtype and high-risk stratification. Due to its behavior during treatment, TK1 can be used by clinicians to monitor treatment response. However, TK1 levels vary in every patient, so determining a baseline level of TK1 for each patient is necessary [137,139].

Fayed et al. evaluated the miR-92a expression in the plasma of 71 children diagnosed with ALL. They reported that miR-92a expression was higher at diagnosis, decreased during remission after maintenance, and increased again during relapse (FC mean ± SD: 17.89 ± 7.9, 7.13 ± 0.93, and 14.72 ± 3.8, respectively). They also validated the diagnostic utility of miR-92a via ROC curve analysis (cut-off = 8.77, AUC = 0.755, sensitivity = 4.5%, and specificity = 100%). In addition, miR-92a correlated with the total leukocytic count (r = 0.547, *p* < 0.0001) and the percentage of blast cells in bone marrow (r = 0.588, *p* < 0.0001) [6]. Ohyashiki et al. evaluated miR-92a in the PBMCs and plasma of 91 patients with AL and found a correlation between the ALL patients with higher levels of miR-92a expression in PBMCs samples and shorter survival (*p* = 0.061) [75]. Overall, miR-92a may be used to predict a poor outcome and treatment response in patients with ALL, and it may to play a role as oncomiR in ALL. Because of the variation in levels during treatment, miR-92a could also help predict the response to treatment, specifically relapse, at diagnosis. Moreover, its correlation with risk factors and OS could enhance its prognostic relevance in patients with ALL [6,75].

Yokota et al., analyzed serum CD44 levels in 25 patients with AL, 12 patients carrying with bacterial infectious diseases, and 13 healthy controls, before and after treatment. Patients with bacterial infection and AL had higher levels before treatment than controls (range: 450–622.5 vs. 313.6–6629.3 vs. 86.4–451.8 ng/mL). There was a trend for decreased CD44 levels after treatment in patients with AL, specifically in patients with ALL. There was an increase in those patients who had a relapse (CD44 > 500 ng/mL after treatment), even when it was not clinically apparent. Lastly, the authors found a correlation between CD44 levels and absolute numbers of leukemic cells in peripheral blood [140]. Takeuchi et al. measured serum levels of CD44 levels in peripheral blood samples of 14 children diagnosed with AL before and after treatment (chemotherapy protocol consisted of induction with vincristine, prednisone, L-asparaginase, and anthracyclines, and consolidation with a high dose of methotrexate and araC) at 6–12 months after diagnosis. They documented decreased CD44 levels after therapy in patients with ALL (range: 1343 ± 5399 vs. 545 ± 63 ng/mL) and AML (range: 1629 ± 953 vs. 394 ± 95 ng/mL, *p* < 0.01). In three patients who relapsed, the CD44 levels increased again [141]. It is assumed that the discoveries are attributed because CD44 is released from malignant cells, which reflect the tumor mass in AL. Thus, CD44 levels are a tumor marker in AL that indicates the clinical status, and its measurement may help monitor its development. Moreover, as increased levels after treatment imply relapse, it can also define optimum chemotherapy [140,141].

Survivin expression and protein levels have been evaluated to determine their ability to measure the response to therapy in a diverse cohort of patients. Mohammadi et al. selected 85 pediatric Irani patients with BCP-ALL and classified them according to their stage of treatment and response to it [70]. They measured surviving mRNA expression in PBMCs to determine changes in patients who did not respond to treatment and those at the recurrence stage (*p* < 0.001). In general, survivin expression was more elevated in patients than controls (*p* < 0.05); ROC curve analysis revealed an AUC of 0.8562 (95% CI: 0.7131–0.9993). Moreover, the survivin protein levels were elevated in the cohort of patients [70]. These findings parallel those reported by Ahmed et al., who evaluated the survivin concentration in the serum of 30 patients with ALL before receiving treatment and after 4 weeks of induction. The authors reported that at diagnosis, survivin levels in patients with ALL were higher than in the control group (range: 28.0 ± 11.2 vs. 3.3–14.8 pg/mL; *p* = 0.00), showing a considerable reduction in survivin levels after therapy. The survivin cut-off based on ROC curve analysis was 15.18 pg/mL (AUC = 0.985, sensitivity 91.7%, specificity 100%, *p* < 0.01). The percentage of patients with ALL above the cut-off value was remarkably higher before therapy than after therapy. Finally, the authors found a correlation between blast in the bone marrow and the blast count in the peripheral blood. That association with risk factors suggests that survivin enhances aggressive behavior. Moreover, the ROC curves for serum survivin to discriminate between patients with ALL and the control group revealed good sensitivity and specificity [3]. Taken together, survivin mRNA and protein levels are potential cancer markers that could diagnose and determine the severity of the disease and response to treatment for ALL and BCP-ALL [3,70]. 

Three adhesion molecules have been evaluated in the serum of peripheral blood samples in children with newly diagnosed with ALL, with two additional samples 6 months after starting treatment, 6 months after treatment, and during relapse. The intercellular adhesion molecule (ICAM-1), vascular cell adhesion molecule-1 (VCAM-1), and E-selectin expression levels were higher in patients with ALL than controls. In the same way, five patients with concentrations of ICAM-1 > 800ng/mL, VCAM-1 > 300ng/mL, and E-selectin > 200 ng/mL relapsed. These values can be used as markers for the severity of ALL. However, the levels of circulating soluble adhesion molecules can depend on the synthesis of their receptor or their capacity to bind ligands expressed on vascular endothelium. Therefore, sE-selectin, sVCAM-1, and sICAM-1 are potential indicators of the trend to later relapse and for monitoring ALL activity [142].

#### 4.2.5. MRD

In the pediatric population, MRD evaluation is routinely used. It refers to the presence of leukemic cells below the threshold of detection by conventional morphological methods [12,143]. It is essential to evaluate MRD to determine the sequential steps after treatment (chemotherapy, immunotherapy, or radiotherapy) [12,22]. Specifically, MRD by flow cytometry, one of the most widely used studies to evaluate MRD, requires BMP; children are put under general anesthesia (GA) and undergo BM aspiration for MRD follow-up 2–5 times following their diagnosis [12,144,145]. This is because if little residual leukemia is left, it is most reliably and abundantly found in the bone marrow, general anesthesia and BMP, together with preparations and related issues, put quite a burden on children, their families, and hospital staff [146]. 

Circulating cell-free nucleic acids refer to the nucleic acid fragments in plasma or serum released into the blood due to necrosis, apoptosis, autophagy, or mitosis [50,147]. The DNA quantity and integrity index are altered in patients with malignant disease, and these changes are related to the tumor stage [50,147]. Some studies have demonstrated the utility of cf-DNA as a biomarker for early cancer detection, progression, treatment response, drug resistance, analyzing acquired resistance, and guiding therapy in several cancers [148,149]. A study conducted by Gao et al. analyzed the concentration and integrity of cf-DNA in plasma peripheral blood samples from 60 patients with AL. The researchers found that the concentration of cf-DNA was higher in patients compared to controls, (range: 0.81–34.90 vs. 0.22–0.853). The diagnostic utility of cf-DNA showed an AUC of 0.79, a sensitivity of 65%, and a specificity of 90% (at a threshold of 7.05 ng/mL). The authors used the same approach to evaluate the cf-DNA integrity index, which was higher in patients with AL (AUC = 0.88, sensitivity of 78%, and specificity of 90%; threshold = 0.32). They also evaluated the cf-DNA integrity index for the detection of MRD in eight patients, with results showing that the index was higher at diagnosis (median 0.46–0.76), decreased at CR (range 0.23–0.50), and increased again at relapse (range 0.38–0.70) (*p* < 0.01). These findings suggest that the cf-DNA integrity index can be used to monitor disease progression in AL patients [50].

Researchers examined the activity of antibodies against 9-0AcSGs (anti 9-OAcSGs) in serum samples from the peripheral blood from children with ALL divided into two groups of patients with different treatment schemes: Group I, UK ALL X; and Group II, UK ALL 97. The authors reported that titers of anti 9-OAcSGs progressively decreased in both groups during chemotherapy (OD_405_ nm of 0.77 ± 0.12 and 0.68 ± 0.16 at consolidation, 0.4 ± 0.08 and 0.38 ± 0.06 at maintenance, and 0.28 ± 0.07 and 0.29 ± 0.06 at follow-up). These findings were further supported by creating a standard curve to assess the accuracy of the assays; the sensitivity and specificity were 98.92% and 92.1%, respectively, for Group I; and 96.77% and 95.91%, respectively, for Group II. Finally, anti-9-OAcSG levels increased 17 weeks before clinical manifestations, presumably because of the antigen’s high immunogenicity. Hence, anti-9-OAcSG can be considered a method to assess MRD and to monitor ALL status [65]. 

Rzepiel et al. evaluated the expression of 46 miRNAs in platelet-free blood plasma samples of 15 de novo and 5 relapsed patients with ALL at diagnosis, and at days 8, 15, and 33 of the ALL IC-BFM 2009 trial protocol. From the 46 selected miRNAs, 19 were significantly dysregulated from controls. Of these, four miRNAs decreased significantly at induction: miR-128-3p (FC = 4.52, *P* = 3.48 × 10^−9^), miR-181a-5p (FC = 2.46, *p* = 2.11 × 10*^−3^*), miR-181b-5p (FC = 3.46, *P* = 4.73 × 10^−5^), and miR-222-3p (FC = 2.68l, *p* = 2.15 × 10^−4^). Of these miRNAs, miR-181b-5p, and miR-128-3p showed the best correlation with cytogenetic subgroups. Then, they performed ROC analysis to determine the accuracy of these miRNAs to differentiate patients with high and low MRD. miR-128-3p, miR-222-3p, miR-181b-5p, and miR-181a-5p had an AUC of 0.914, 0.787, 0.707, and 0.600, respectively. Because of the change in normalized expression from day 0 to8 of miR-128-3p, miR-181b-5p appears to be the best MRD biomarker for ALL [146].

## 5. Conclusions

**Figure 4:**
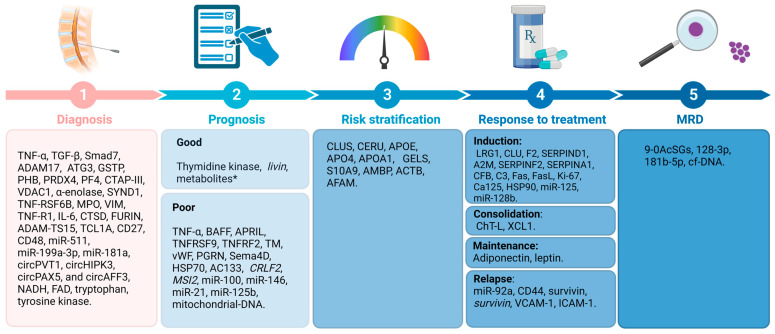
Here is a summary of the biomarkers that were discussed in the text for B-cell precursor acute lymphoblastic leukemia and their uses according the disease stage: * LysoPC(16:0); LysoPC(18:0); PC(P-18:1(9Z)/0:0); 3-Decaprenyl-4-hydroxybenzoic acid; DG(24:1(15Z)/22:5(4Z, 7Z,10Z,13Z,16Z)/0:0); LysoPC(18:2(9Z,12Z)); PE(P-19:1(12Z)/0:0); LysoPC(18:1(11Z)); PC(16:0/0:0)[rac]; PC(18:1(9Z)/18:4(6Z,9Z, 12Z,15Z)); PI(20:5(5Z,8Z,11Z,14Z,17Z)/15:1(9Z)); LysoPE(0:0/16:0); LysoPE(18:1(11Z)/0:0); LysoPc(15:0); LysoPC(20:5(5Z,8Z,11Z,14Z,17Z)); LysoPC(22:6(4Z,7Z,10Z,13Z,16Z,19Z)); LysoPC(17:0); LysoPC(22:5(7Z, 10Z,13Z,16Z,19Z)); PC(15:0/251 0:0); 11(Z),14(Z)-eicosadienoic acid; PA(22:0/0:0); PE(14:0/15:0); PG(20:1(11Z)/0:0); PG(P-20:0/0:0); uric acid; chenodeoxycholic acid glycine conjugate; LysoPE(22:4(7Z, 10Z,13Z,16Z)/0:0); and LysoPE(20:4(8Z,11Z,14Z,17Z)/0:0).

Although ALL treatment has a success story in pediatric oncology, the results in adults continue to lag behind. ALs are rapidly progressive, with death often occurring in a few weeks to a few months after diagnosis in untreated patients due to abnormal hematopoietic function and an inadequate immune response [74]. Further, a BMP, an expensive and quite invasive procedure, is necessary for an adequate diagnosis. Thus, circulating biomarkers are an excellent alternative that would reduce cost and invasiveness. We have compiled a list of 182 biomarkers that can aid in diagnosis: 118 for prognosis, 14 for risk stratification, 30 for determining the response to treatment, and 5 for MRD (Supplementary Table S1). Specific biomarkers, including TNF-α, leptin, miR-100, miR-511, and cf-DNA, are helpful for multiple disease states (Figure 3). Their potential usefulness is strengthened by multiple reports in different cohorts of patients, giving them a relatively broader endorsement (Figure 4). 

Ongoing research has attempted to identify novel molecules for diagnostic and prognostic biomarkers to increase the specificity and sensitivity of non-invasive procedures to determine and classify the disease and, subsequently, to improve the clinical outcome of patients. However, there is still much work to do. In this review, we have confronted many obstacles regarding gathering information about biomarkers research. Many of the studies we mentioned require larger samples and analytical validation before the biomarkers could be used for in a clinical context. Moreover, only a few studies on prognostic biomarkers compared groups of patients to different treatment schemes, which can affect the clinical outcome. 

Because many underlying mechanisms may affect the biomarker results, it is crucial to look into their role in leukemogenesis and to discard any potential bias. Moreover, by studying their role in cancer biology, it is possible to determine whether the biomarkers are clinically useful or could potentially serve as therapeutic targets for BCP-ALL. Nonetheless, an individual biomarker candidate might be specific and sensitive enough for a particular stage or molecular etiology. We concluded that combinations of many markers screened for sensitivity and specificity could achieve a higher diagnostic and prognostic value for routine clinical practice. To improve diagnostic and prognostic approaches for patients with BCP-ALL, we encourage the scientific community to keep researching circulating biomarkers and to validate the biomarkers that have already been reported.

## Figures and Tables

**Table 1:** ROC values of diagnostic biomarkers for BCP-ALL.

Biomarker	Blood Sample	Leukemia Type	Area under the ROC Curve	Sensitivity (%)	Specificity (%)	PPV (%)	NPV (%)	Reference
Smad 7	Serum	ALL	0.81	63	100	100	73	
TGF-β1	Serum	ALL	0.79	57	93	89.5	68	[16]
Smad 7 TGF-β1 miR-181a	Serum	ALL	-	100	93	93.7	100	
IGF-I	Serum	ALL	-	60.6	73.3	-	-	[64]
IGF-II	Serum	ALL	-	72.2	73.3	-	-	
IGFBP-2	Serum	ALL	-	72.2	86.7	-	-	
IGFBP-3	Serum	ALL	-	93.9	93.9	-	-	
Anti-9-0AcSGs	Serum	ALL-GI	-	98.9	92.1	96.8	97.2	[65]
Anti-9-0AcSGs	Serum	ALL-GI	-	96.8	95.9	96.8	95.9	
PF4 CTAP-II	Serum	ALL	-	91.8	90	-	-	[7]
C3f	Serum	AL	0.99	97	100	-	-	[45]
TNF-α	Serum	ALL	0.94	91.7	100	-	-	[3]
Survivin	Serum	ALL	0.98	90	80	-	-	
p53	Serum	AL	0.8	52	100	-	-	[66]
EGFR	Serum	AL	0.93	73.9	95.8	-	-	
Pseudouridine	Serum	ALL	-	90	97.5	-	-	[67]
ADAM 17	Plasma	BCP-ALL	0.98	100	100	-	-	[68]
ATG3	Plasma	BCP-ALL	0.95	100	100	-	-	
AC133 *	Whole blood	ALL	-	100	100	100	100	[69]
miR-181a	Serum	ALL	0.93	86.5	93.3	92.8	87.5	[16]
miR-146a	Plasma	BCP-ALL	1	100	100	-	-	[9]
mRNA Survivin	Whole blood	BCP-ALL	0.85	95	95	-	-	[70]
mRNA HLA-G	PBMC	ALL	-	74	100	-	-	[71]
miR-125b-1	Serum	ALL	0.85	83.7	100	-	-	[72]
miR-203	Serum	ALL	0.87	97.7	87	-	-	
miR-100	PBMC	ALL	0.87	82.7	100	-	-	[15]
miR-196a	PBMC	ALL	0.537	46.6	100	-	-	
miR-146a	PBMC	ALL	1	100	100	-	-	
miR-511	Plasma	BCP-ALL	1	100	100	1	1	[73]
miR-34a	Plasma	BCP-ALL	0.98	92	100	1	0.70	
miR-22	Plasma	BCP-ALL	0.91	79	100	1	0.54	
miR-26a	Plasma	BCP-ALL	0.91	79	100	1	0.47	
miR-221	Plasma	BCP-ALL	0.92	83	100	1	0.54	
miR-223	Plasma	BCP-ALL	0.93	89	100	1	0.64	
miR-21	PBMC	ALL	0.565	44	55	-	-	[74]
miR-26	PBMC	ALL	0.464	54	50	-	-	
miR-148a	PBMC	ALL	0.719	74	79	-	-	
miR-133b	PBMC	ALL	0.669	70	60	-	-	
miR-24	PBMC	ALL	0.785	72	81	-	-	
miR-92a	PBMC and plasma	ALL	0.99	-	-	-	-	[75]
miR-92a	Plasma	ALL	0.755	41.5	100	100	36.7	[6]
miR-638	Plasma	ALL	0.86	54.7	100	100	42.9	
miR-125b	PBMC	ALL	0.99	98	96.7	-	-	[76]
mRNA-Bcl-2	PBMC	ALL	0.9	96.7	70	-	-	
miR-128b	PBMC	ALL	-	75	87.5	-	-	[77]
cf-DNA levels	Plasma	ALL, AML	0.79	65	100	-	-	[50]
cf-DNA integrity	Plasma	ALL, AML	88	78	90	-	-	

ROC: Receiver operating characteristic; PPV: positive predictive value; NPV: negative predictive value. ALL: acute lymphoblastic leukemia; BCP-ALL: B-cell precursors ALL; AML: acute myeloid leukemia; GI: Group I; GII: Group II; PBMC: peripheral blood mononuclear cell. * Value of AC133 expression to predict cases of poor prognosis.

## Supplementary Materials

The following supporting information can be downloaded at: https://www.mdpi.com/article/10.3390/cancers15164186/s1, Table S1: Circulating Biomarkers for BCP-ALL. References [95,150,151,152,153,154,155,156,157,158,159,160,161,162] are cited in the supplementary materials.
