# Supplementary material for: Circulating Biomarkers Associated with the Diagnosis and Prognosis of B-Cell Progenitor Acute Lymphoblastic Leukemia
Source: Cancers (Basel). 2023 Aug 20;15(16):4186. doi: 10.3390/cancers15164186 (PMC10453581; doi:10.3390/cancers15164186)
Supplement: Supplementary file 1 [file cancers-15-04186-s001.zip › cancers-2541670-supplementary.pdf]

Circulating Biomarkers Associated with the Diagnosis and Prognosis of B-Cell Progenitor Acute Lymphoblastic Leukemia. Álvarez-Zúñiga, C.D.; Garza-Veloz, I.; Martínez-Rendón, J.; Ureño-Segura, M.; Delgado-Enciso, I.; Martinez-Fierro, M.L. *Cancers* **2023.**

Table S1: Circulating Biomarkers for BCP-ALL.

Marker	Association	Blood component	Expression	P	Method	Finding	Ref.
p53	AL	Serum	Up	<0.05	ELISA	Diagnosis and differentiation between adult ALL and AML.	[66]
EGFR	AL	Serum	Up	<0.001	ELISA		
TNF-α	BCP-ALL	Plasma	Up	0.024	ELISA	Diagnosis and useful to asses childhood BCP-ALL.	[63]
	ALL	Serum	Up	0	ELISA	Diagnostic and prognostic biomarker in adults with ALL.	[3]
	BCP-ALL	Serum	Up	>0.05	ELISA	Difference from others hematological disease.	[62]
Leptin	BCP-ALL	Plasma	Up	<0.001	Flow cytometry	Diagnosis, correlating the disease stage and the responsiveness to chemotherapy or a new target for childhood ALL therapy.	[134]
	ALL	Serum	High	-	ELISA	Prognostic marker, leukemia stratification in adults with ALL and AML.	[135]
Adiponectin	ALL	Serum	Low	-	ELISA	Prognostic marker, leukemia stratification in adults with ALL and AML.	[135]
	BCP-ALL	Plasma	Down	<0.0001	Flow cytometry	Diagnosis, correlating the disease stage and the responsiveness to chemotherapy or a new target for childhood ALL therapy.	[134]
Resistin	BCP-ALL	Plasma	Up	<0.001	Flow cytometry	Diagnosis, correlating the disease stage and the responsiveness to chemotherapy or a new target for childhood ALL therapy.	[134]
Fragment of C3-8137	ALL	Serum	Up	$5.35x10^{-5}$	Specific antibody	Potential biomarker for diagnosis of pediatric ALL, and distinguish between AML.	[4]
Fragment of C3-8937	ALL	Serum	Up	$5.13x10^{-5}$	Specific antibody		
C3	BCP-ALL	Serum	Up	<0.05	Lectin affinity chromatography	Candidate biomarker for early diagnosis and response to treatment of childhood BCP-ALL.	[124]
Tyrosine Kinase	ALL	Plasma	Down	<0.05	Fluorescence spectral	Diagnostic and diffused malignancy indicator of childhood ALL.	[8]
Thymidine kinase	BCP-ALL, TCP-ALL, AML	Serum	Up	0.001	Two-step indirect immunoassay modified by chemiluminescence	Diagnostic biomarker for B-ALL.	[139,137]
				<0.001	Immunoassay with specific monoclinal antibody.	Diagnostic, following up and response to treatment biomarker to ALL.	
CD44	BCP-ALL	Plasma	Up	≤0.01	FT-FCM	Potential diagnosis of adult BCP-ALL and new targets for therapy.	[49]
	ALL	Serum	Up	<0.01	ELISA	Reliable tumor marker for adult AL and pediatric ALL	[140,141]
BAFF	ALL	Plasma	Up	<0.05	ELISA	Diagnostic and progression marker for childhood ALL	[150,151]
	ALL	Serum	Up	<0.0001	ELISA	Diagnostic and prognostic markers for adult ALL.	[94]
APRIL	ALL	Plasma	Up	<0.05	ELISA	Diagnostic and progression marker for childhood ALL	[150,151]
	ALL	Serum	Up	<0.0001	ELISA	Diagnostic and prognostic markers for adult ALL.	[94]

PF4	ALL	Serum	Down	1.54x10^{-7}	Specific antibody	Potential biomarker for diagnosis of pediatric ALL, and distinguish between AML.	[7]
TM	ALL	Plasma	Up	<0.001	ELISA	Prognostic marker in childhood ALL.	[99]
	ALL	Plasma	Up	0.0001	ELISA		[98]
vWF	ALL	Plasma	Up	<0.001	ELISA	Prognostic marker in childhood ALL.	[99]
	ALL	Plasma	Up	0.025	ELISA		[98]
	AL	Plasma	Up	6x10^{-85}	PEA	Clinical prognostic markers and treatment effect of adult AL.	[78]
Survivin	ALL	Serum	Up	0	ELISA	Diagnosis and prognostic of adult ALL.	[3]
	BCP-ALL	PBMC	Up	<0.0001	Western Blot	Potential marker to specify prognosis in childhood BCP-ALL.	[70]
Tryptophan	ALL	Plasma	Down	<0.05	Fluorescence spectral	Diagnostic and diffused malignancy indicator of childhood ALL.	[8]
Coenzyme NADH	ALL	Plasma	Down	<0.05			
Coenzyme FAD	ALL	Plasma	Up	<0.05			
CTAP-III	ALL	Serum	Down	7.19x10^{-8}	Specific antibody	Potential biomarker for diagnosis of pediatric ALL, and distinguish between AML.	[7]
GSTP	ALL	Whole blood	Up	<0.01	PMF	Potential diagnostic biomarker and drug target of childhood ALL.	[4]
PHB	ALL	Whole blood	Up	<0.01	PMF		
60S acidic ribosomal protein P0	ALL	Whole blood	Down	<0.01	PMF		
PNPO	ALL	Whole blood	Down	<0.01	PMF		
TPI-1	ALL	Whole blood	Down	<0.05	PMF		
PRDX4	ALL	Whole blood	Down	<0.01	PMF		
Cytoplasmic actin	ALL	Whole blood	Down	<0.01	PMF		
Hypothetical protein FLJ26567	ALL	Whole blood	Down	<0.01	PMF		
ADAM17	BCP-ALL	Plasma	Up	8x10^{-5}	Western blot	Diagnostic biomarker and develop therapeutic target for adult BCP-ALL.	[68]
ATG3	BCP-ALL	Plasma	Up	2x10^{-6}	Western blot		
CD19	BCP-ALL	Plasma	Up	≤0.05	FT-FCM	Potential diagnosis of adult BCP-ALL and new targets for therapy	[49]
CD24	BCP-ALL	Plasma	Up	≤0.05	FT-FCM		
CD29	BCP-ALL	Plasma	Up	≤0.01	FT-FCM		
CD105	BCP-ALL	Plasma	Up	≤0.01	FT-FCM		
CD146	BCP-ALL	Plasma	Up	<0.05	FT-FCM		
CD9	BCP-ALL	Plasma	Up	≤0.05	FT-FCM		
CD63	BCP-ALL	Plasma	Up	≤0.01	FT-FCM		
CD81	BCP-ALL	Plasma	Up	≤0.05	FT-FCM		
HLA-DR	BCP-ALL	Plasma	Up	≤0.06	FT-FCM		
Smad7	ALL	Serum	Up	<0.01	qRT-PCR	Potential diagnostic marker for childhood ALL	[16]
α-enolase	BCP-ALL	Serum	Up	<0.05	ELISA	Potential diagnostic, prognostic biomarkers and therapeutical targets.	[14]
Anti-9-0AcSGs	ALL	Serum	Up	<0.0001	ELISA	Potential diagnostic biomarker for childhood ALL.	[65]
TGF-β1	ALL	Serum	Down	<0.01	qRT-PCR	Potential diagnostic marker for childhood ALL	[16]

Semaphorin 4D	AML, ALL	Plasma	Up	<0.01	ELISA	Target for leukemia diagnosis and treatment	[105]
	BCP-ALL	PBMC	Up	<0.01	Western blot	Potential biomarker for pediatric BCP-ALL diagnosis and prognosis	[106]
VEGF	ALL	Serum	Down	<0.001	ELISA	Predictor of progression of childhood ALL.	[152]
sL-selectin	ALL	Serum	Up	<0.05	ELISA	Diagnostic and detection of relapse of childhood ALL.	[153]
BCMA	ALL	Plasma	Up	<0.05	ELISA	Diagnostic and prognostic markers for adult ALL.	[94]
TACI	ALL	Plasma	Up	<0.05	ELISA		
TRAIL	ALL	Serum	Down	<0.0001	ELISA		
CLUS	BCP-ALL	Plasma	Down	<0.01	Western blot.	Potential diagnostic and prognostic biomarker for pediatric -ALL.	[110]
CERU	BCP-ALL	Plasma	Down	<0.01	Western blot.		
APOE	BCP-ALL	Plasma	Down	<0.01	Western blot.		
APOA4	BCP-ALL	Plasma	Up	<0.01	Western blot.		
APOA1	BCP-ALL	Plasma	Up	<0.01	Western blot.		
AMBP	BCP-ALL	Plasma	Up	<0.01	Western blot.		
ACTB	BCP-ALL	Plasma	Down	<0.01	Western blot.		
AFAM	BCP-ALL	Plasma	Down	<0.01	Western blot.		
LRG1	BCP-ALL	Serum	Up	<0.05	Lectin affinity chromatography	Candidate biomarker for early diagnosis and response to treatment of childhood BCP-ALL.	[124]
CLU	BCP-ALL	Serum	Up	<0.05			
F2	BCP-ALL	Serum	Up	<0.05			
SERPIND1	BCP-ALL	Serum	Up	<0.05			
A2M	BCP-ALL	Serum	Up	<0.05			
SERPINF2	BCP-ALL	Serum	Up	<0.05			
SERPINA1	BCP-ALL	Serum	Up	<0.05			
CFB	BCP-ALL	Serum	Up	<0.05			
ADA	ALL	Serum	Up	<0.001	Electrophoresis	Diagnose childhood ALL and monitor therapies.	[154]
ADA1	ALL	Serum	Up	<0.001	Electrophoresis		
IGF-I	ALL	Serum	Down	0.08	ELISA	Support diagnosis and follow-up of children with ALL	[64]
IGF-II	ALL	Serum	Down	0.79	ELISA		
IGFBP-2	ALL	Serum	Up	0.22	ELISA		
IGFBP-3	ALL	Serum	Down	0.9	ELISA		
cKI-67	ALL	Plasma	Up	<0.001	ECL	Diagnostic, prognostic biomarker in adult ALL.	[126]
cCD 33	ALL, AML	Plasma	High	0.03*	IFC	Poor prognosis in adult patients with ALL.	[155]
CD56	BCP-ALL	Whole blood	In vivo	0.006	Flow cytometry	Poor prognosis in adult BCP-ALL	[156]
CD200	BCP-ALL	Whole blood	In vivo	0.003	Flow cytometry		
Ca 125	ALL, ANLL	Serum	High	0.001***	ELISA	Therapeutic response in children with AL.	[127]
sI-CAM-1	ALL	Serum	Up	<0.001	ELISA	Response to treatment and early detection of relapse of childhood ALL.	[142]
sVCAM	ALL	Serum	Up	<0.001	ELISA		

sE-selectin	ALL	Serum	Up	<0.001	ELISA		
TNFRS2	ALL	Plasma	Up	<0.34	ELISA	Poor prognostic biomarker for ALL.	[96]
	ALL	PMNC	Up	<0.081	ELISA		
TNFRS9	ALL	Plasma	Up	<0.15	ELISA		
	ALL	PMNC	Up	<0.42	ELISA		
SYND1	AL	Plasma	Up	$7.2x10^{-71}$	PEA	Clinical prognostic markers and treatment effect of adult AL.	[78]
TNFRSF6B	AL	Plasma	Up	$2.2x10^{-71}$	PEA		
MPO	AL	Plasma	Up	$5.9x10^{-50}$	PEA		
VIM	AL	Plasma	Up	$3.6x10^{-45}$	PEA		
TNF-R1	AL	Plasma	Up	$1.2x10^{-42}$	PEA		
IL-6	AL	Plasma	Up	$3.6x10^{-41}$	PEA		
CTSD	AL	Plasma	Up	$1.6x10^{-40}$	PEA		
ADAM-TS 15	AL	Plasma	Low	$3.4x10^{-40}$	PEA		
FURIN	AL	Plasma	Up	$6x10^{-40}$	PEA		
HSP70	ALL, AML	Plasma	Up	<0.05	Electrochemiluminescence	Potential adverse prognostic biomarker of adult ALL and AML.	[107]
PGRN	ALL	Serum	Up	CIR:0.008	ELISA	Marker of predictor ALL and target therapy.	[101]
				DFS:0.029			
sFASL	ALL	Serum	Up	Survival:0.0018	ELISA	Marker associated with favorable response to therapy in childhood ALL.	[125]
				CRD: 0.0013			
XCL1	ALL	Serum	Up	0.01	ELISA	Marker related with higher survival in adult ALL patients.	[21]
Statin	ALL	Whole blood	Up	0.05	Mab	Additional useful marker for prognosis in childhood ALL.	[46]
HSP90	ALL	Serum	Down	0.192	ELISA	Marker related to poor response to steroids in children with ALL.	[130]
Proteasome ChT-L	ALL	Plasma	Up	0.006	Fluorogenic peptide assay	Additional marker in monitoring the therapy response in childhood ALL.	[132]
AC133	AL	Whole blood	Up	<0.05	Flow cytometry	Prognostic marker to high resistance to chemotherapy and short survival in childhood AL.	[69]
mR-223-3p	AL	Serum	Up	0.0044	qRT-PCR	Diagnostic biomarker of childhood ALL.	[15]
mRNA Survivin	BCP-ALL	Whole blood	Up	<0.05	qRT-PCR	Potential diagnostic marker for childhood BCP-ALL.	[70]
mRNA LEF-1	BCP-ALL	Whole blood	Up	OS: 0.005	qRT-PCR	Adverse prognostic significance for adult BCP-ALL.	[20]
mRNA-BAFF	ALL	PBMC	UP	<0.05	RT-PCR	Diagnostic and progression marker for childhood ALL.	[150]
mRNA-APRIL	ALL	PBMC	UP	<0.05	RT-PCR		
mRNA-BCMA	ALL	PBMC	UP	<0.05	RT-PCR	Diagnostic and progression marker for childhood ALL.	[150,151]
mRNA-TACI	ALL	PBMC	UP	<0.05	RT-PCR	Diagnostic and progression marker for childhood ALL.	
mRNA Livin	ALL	PBMC	Up	OS:0.05	qRT-PCR	Potential prognostic marker for childhood ALL.	[111]
mRNA LRP	ALL	PBMC	Up	<0.05	RT-PCR	Marker associated with early response and complete remission state post induction in pediatric ALL.	[157]

mRNA HLA-G	ALL	PBMC	High	<0.01	RT-PCR	Prognostic tumor marker and to monitor disease state and improvement in adult ALL.	[71]
mRNA Bcl-2	ALL	PBMC	Down after induction	<0.001	qRT-PCR	Potential prognostic marker and monitor response to treatment for childhood ALL.	[76]
mRNA MS12	BCP-ALL	Whole blood	Up	OS: 0.018	RT-PCR	Unfavorable prognostic biomarker for adult BCP-ALL.	[27]
mRNA CRLF2	BCP-ALL	Whole blood	Up	OS: 0.0038	Q-PCR	Unfavorable prognostic biomarker of long-term outcome for adult BCP-ALL.	[112]
miR-181a	ALL	Serum	Down	<0.01	qRT-PCR	Potential diagnostic marker for childhood ALL	[16]
miR-181b	ALL	Whole blood	Up	<0.001	qRT-PCR	Diagnosis of childhood ALL.	[158]
miR-181c	ALL	Whole blood	Up	<0.001	qRT-PCR		
miR-181b-5p	ALL	Plasma	Up	<0.001	qRT-PCR and TLDA	Detection of minimal residual leukemia.	[146]
miR-196	ALL	PBMC	Up	5.00×10^{-6}	qRT-PCR	Potential diagnostic biomarker and therapeutic targets for childhood ALL.	[116]
miR-196a	ALL	PBMC	Down	0.028	qRT-PCR	Diagnostic biomarker of childhood ALL.	[15]
miR-10b	ALL	PBMC	Up	5.00×10^{-6}	qRT-PCR	Potential diagnostic biomarker and therapeutic targets for childhood ALL.	[116]
miR-15b	ALL	PBMC	Up	5.00×10^{-6}	qRT-PCR		
miR-20b	ALL	PBMC	Down	5.00×10^{-5}	qRT-PCR		
miR-22	ALL	PBMC	Down	1.50×10^{-5}	qRT-PCR		
miR-23a	ALL	PBMC	Up	5.00×10^{-6}	qRT-PCR		
miR-25	ALL	PBMC	Down	$4.00x10^{-5}$	qRT-PCR		
miR-29a	ALL	PBMC	Down	1.00×10^{-5}	qRT-PCR		
miR-30c	ALL	PBMC	Down	1.00×10^{-5}	qRT-PCR		
miR-30e-3p	ALL	PBMC	Down	$5.00x10^{-5}$	qRT-PCR		
miR-100	ALL	PBMC	Down	$4.00x10^{-5}$	qRT-PCR		
miR-100	ALL	Whole blood, plasma.	Up	<0.001	qRT-PCR	Potential biomarker for childhood acute leukemia	[117]
	ALL	PBMC	Up	0.007	qRT-PCR	Diagnostic biomarker of childhood ALL.	[15]
	ALL	PBMC	Up	0.002	qRT-PCR	Biomarker associated with resistance to vincristine in childhood ALL.	[118]
miR-101	ALL	PBMC	Down	$5.00x10^{-6}$	qRT-PCR	Potential diagnostic biomarker and therapeutic targets for childhood ALL.	[116]
miR-126	ALL	PBMC	Down	$6.50x10^{-5}$	qRT-PCR		
miR-126	ALL	PBMC	Up	0.046	qRT-PCR	Biomarker associated with resistance to vincristine in childhood ALL.	[118]
miR-129	ALL	PBMC	Up	$5.00x10^{-5}$	qRT-PCR	Potential diagnostic biomarker and therapeutic targets for childhood ALL.	[116]
miR-133b	ALL	PBMC	Up	5.00×10^{-6}	qRT-PCR		
miR-146b	ALL	PBMC	Down	$5.00x10^{-6}$	qRT-PCR		
miR-146a-3p	AL	Serum	Down	0.0012	qRT-PCR	Early detection of pediatric acute leukemia	[159]
miR-146a	ALL	PBMC	Up	<0.0001	qRT-PCR	Diagnostic biomarker of childhood ALL.	[15]
	ALL	Plasma	Up	<0.001	qRT-PCR	Predictive marker for diagnosis and prognosis of adult and pediatric ALL	[9]

miR-155	ALL	PBMC	Up	5.00×10^{-6}	qRT-PCR	Potential diagnostic biomarker and therapeutic targets for childhood ALL.	[116]
miR-193b	ALL	PBMC	Down	5.00×10^{-6}	qRT-PCR		
miR-216	ALL	PBMC	Down	$2x10^{-5}$	qRT-PCR		
miR-217	ALL	PBMC	Up	5.00×10^{-6}	qRT-PCR		
miR-299-3p	ALL	PBMC	Down	$2.50x10^{-5}$	qRT-PCR		
miR-302b	ALL	PBMC	Up	5.00×10^{-6}	qRT-PCR		
miR-302d	ALL	PBMC	Down	5.00×10^{-6}	qRT-PCR		
miR-325	ALL	PBMC	Down	5.00×10^{-6}	qRT-PCR		
miR-330	ALL	PBMC	Up	5.00×10^{-6}	qRT-PCR		
miR-362	ALL	PBMC	Up	5.00×10^{-6}	qRT-PCR		
miR-368	ALL	PBMC	Up	5.00×10^{-6}	qRT-PCR		
miR-696-5p	ALL	PBMC	Up	$4.50x10^{-5}$	qRT-PCR		
miR-374	ALL	PBMC	Down	5.00×10^{-6}	qRT-PCR		
miR-425-5p	ALL	PBMC	Up	$5.00x10^{-6}$	qRT-PCR		
miR-494	ALL	PBMC	Down	1.00×10^{-5}	qRT-PCR		
miR-501	ALL	PBMC	Up	5.00×10^{-6}	qRT-PCR		
miR-513	ALL	PBMC	Up	$2.50x10^{-6}$	qRT-PCR		
miR-514	ALL	PBMC	Down	5.00×10^{-6}	qRT-PCR		
miR-515-5p	ALL	PBMC	Down	1.00×10^{-5}	qRT-PCR		
miR-517b	ALL	PBMC	Up	5.00×10^{-6}	qRT-PCR		
miR-520	ALL	PBMC	Up	5.00×10^{-6}	qRT-PCR		
miR-532	ALL	PBMC	Down	$7.5x10^{-5}$	qRT-PCR		
miR-576	ALL	PBMC	Up	$2.5x10^{-7}$	qRT-PCR		
miR-585	ALL	PBMC	Up	5.00×10^{-6}	qRT-PCR		
miR-617	ALL	PBMC	Up	$2.5x10^{-5}$	qRT-PCR		
miR-645	ALL	PBMC	Up	$1.5x10^{-5}$	qRT-PCR		
miR-26a	BCP-ALL	Plasma	Down	<0.001	qRT-PCR	Diagnostic biomarker for BCP-ALL.	[73]
miR-34a	BCP-ALL	Plasma	Up	0.015	qRT-PCR		
miR-221	BCP-ALL	Plasma	Down	<0.001	qRT-PCR		
miR-222	BCP-ALL	Plasma	Up	0.004	qRT-PCR		
miR-99a	ALL	Whole blood	Down	<0.001	qRT-PCR	Diagnosis of childhood ALL	[117]
	ALL	PBMC	Up	0.002	qRT-PCR	Biomarker for predicting prognosis and response to treatment for childhood ALL.	[77]
	ALL	PBMC	Up	0.033	qRT-PCR	Biomarker associated with resistance to vincristine in childhood ALL.	[118]
miR-125b-1	ALL	Serum	Up	<0.0001	qRT-PCR	Potential diagnosis marker for childhood ALL	[72]
miR-125b-5p	BCP-ALL	Plasma	Up	<0.01	qRT-PCR and TLDA	Diagnostic of BCP-ALL and detection of residual leukemia	[116]

miR-125b	ALL	PBMC	Up after induction	<0.001	qRT-PCR	Prognostic tumor marker and to monitor disease state and improvement in adult ALL.	[71]
	ALL	PBMC	Down	0.001	qRT-PCR	Biomarker for predicting prognosis and response to treatment for childhood ALL.	[77]
	ALL	PBMC	Up	0.033	qRT-PCR	Biomarker associated with resistance to vincristine in childhood ALL.	[118]
miR-203	ALL	Serum	Down	<0.0001	qRT-PCR	Early detection of pediatric acute leukemia	[159]
	ALL	PBMC	Up	0.041	qRT-PCR	Biomarker associated with resistance to vincristine in childhood ALL.	[118]
miR-142-3p	AL	Serum	Down	0.001	qRT-PCR	Early detection of pediatric acute leukemia	[159]
miR-223	BCP-ALL	Plasma	Down	<0.001	qRT-PCR and TLDA	Diagnostic of BCP-ALL and detection of residual leukemia	[146,73]
miR-511	BCP-ALL	Plasma	Up	0.002	qRT-PCR	Diagnosis, identification of progression markers and assessment of biological and/or therapeutic targets for patients with BCP-ALL.	[73]
miR-638	ALL	Plasma	Up	<0.0001	qRT-PCR	Detection, prognosis and relapse at diagnosis time	[6]
circRNA-AFF2	BCP-ALL	PBMC	Up	0.029	qRT-PCR	Classification, risk stratification and diagnosis of BCP-ALL.	[90]
circRNA-IL4R	BCP-ALL	PBMC	Up	<0.01	qRT-PCR		
circRNA-ZCCHC7	BCP-ALL	PBMC	Up	<0.01	qRT-PCR		
circRNA-AFF3	BCP-ALL	PBMC	Up	<0.01	qRT-PCR		
circRNA-PAX5	BCP-ALL	PBMC	Up	<0.0001	qRT-PCR		
circRNA-PVT1	BCP-ALL	PBMC	Up	0.0002	qRT-PCR		
circRNA-HIPX3	BCP-ALL	PBMC	Up	<0.0001	qRT-PCR		
circRNA-BCL2	BCP-ALL	PBMC	Up	0.0317	qRT-PCR		
circRNA-SETBP1	MLL, BCP-ALL	PBMC	Down	0.0028	qRT-PCR		
circRNA-X	MLL-BCP-ALL	PBMC	Down	0.0472	qRT-PCR		
circRNA-IKZF1	BCP-ALL	PBMC	Down	0.0154	qRT-PCR		
miR-21	ALL	PBMC	Up	<0.05	qRT-PCR	Response to treatment in childhood ALL.	[74,115]
	BCP-ALL	Serum	Up	<0.001	qRT-PCR	Biomarker for risk assessment and unfavorable prognostic in childhood BCP-ALL	[160]
miR-92a	ALL	PBMC	Up	0.0186	qRT-PCR	Prognostic and monitoring adult ALL.	[75]
miR-24	ALL	PBMC	Up	<0.05	qRT-PCR	Response to treatment, therapeutic target and prognostic marker in childhood ALL.	[74]
miR-148a	ALL	PBMC	Up	<0.01	qRT-PCR	Response to treatment in childhood ALL.	
miR-128-3p	ALL	Plasma	Up	<0.001	qRT-PRC and TLDA	Detection of minimal residual leukemia.	[146]
miR-128b	ALL	PBMC	Up	<002	qRT-PCR	Biomarker associated with resistance to vincristine in childhood ALL.	[118]
miR-150	ALL	PBMC	Down	0.049	qRT-PCR	Potential predictor of clinical outcome of ALL.	[161]
miR-629	ALL	PBMC	Up	0.031	qRT-PCR	Biomarker associated with resistance to vincristine in childhood ALL.	[118]
miR-9	ALL	PBMC	Up	0.032	qRT-PCR		
miR-625	ALL	PBMC	Down	0.006	qRT-PCR		
miR-141	ALL	PBMC	Down	0.021	qRT-PCR		

miR-200c	ALL	PBMC	Down	0.001	qRT-PCR		
miR-383	ALL	PBMC	Up	0.037	qRT-PCR		
miR-335	ALL	PBMC	Up	0.033	qRT-PCR		
miR-454	ALL	PBMC	Up	0.017	qRT-PCR		
Mitochondrial DNA	ALL	PBMC	Down	EFS: 0.04	qRT-PCR	A marker of poor prognosis of childhood ALL.	[119]
cf-DNA	ALL	Plasma	Up	<0.001	qRT-PCR	DNA concentrations and mean integrity may be used as biomarker for diagnostic, prognostic and monitoring MDR for ALL.	[50]
Pseudouridine	ALL	Serum	Up	<0.001	HPLC	Diagnostic and prognostic marker of adult ALL.	[67]
Phosphorylcholine	ALL	Serum	Up	<0.05	Ultra-performance liquid chromatography and mass spectrometry	Potential diagnostic and prognostic biomarker for childhood ALL.	[17]
LysoPC (16:0)	ALL	Serum	Up	<0.05			
LysoPC (18:0)	ALL	Serum	Up	<0.05			
PC(P-18:1(9Z)/0:0)	ALL	Serum	Up	<0.05			
3-Decaprenyl-4-hydroxybenzoic acid	ALL	Serum	Up	<0.05			
DG (24:1(15Z)/22:5(4Z,7Z,10Z,13Z,16Z)/0:0)	ALL	Serum	Up	<0.05			
LysoPC (18:2(9Z,12Z))	ALL	Serum	Up	<0.05			
PE(P-19:1(12Z)/0:0)	ALL	Serum	Up	<0.05			
LysoPC (18:1(11Z))	ALL	Serum	Up	<0.05			
PC (16:0/0:0) [rac]	ALL	Serum	Up	<0.05			
PC (18:1(9Z)/18:4(6Z,9Z,12Z,15Z))	ALL	Serum	Up	<0.05			
PI (20:5(5Z,8Z,11Z,14Z,17Z)/15:1(9Z))	ALL	Serum	Up	<0.05			
11(Z),14(Z)-eicosadienoic Acid	ALL	Serum	Up	<0.05			
PA (22:0/0:0)	ALL	Serum	Up	<0.05			
PE (14:0/15:0)	ALL	Serum	Up	<0.05			
PG (20:1(11Z)/0:0)	ALL	Serum	Up	<0.05			
PG(P-20:0/0:0)	ALL	Serum	Up	<0.05			
11(Z),14(Z)-eicosadienoic Acid	ALL	Serum	Up	<0.05			
PA (22:0/0:0)	ALL	Serum	Up	<0.05			
PE (14:0/15:0)	ALL	Serum	Up	<0.05			
PG (20:1(11Z)/0:0)	ALL	Serum	Up	<0.05			
PG(P-20:0/0:0)	ALL	Serum	Up	<0.05			
LysoPE (0:0/16:0)	ALL	Serum	Down	<0.05			
LysoPE (18:1(11Z)/0:0)	ALL	Serum	Down	<0.05			
LysoPc (15:0)	ALL	Serum	Down	<0.05			
LysoPC (20:5(5Z,8Z,11Z,14Z,17Z))	ALL	Serum	Down	<0.05			
LysoPC (22:6(4Z,7Z,10Z,13Z,16Z,19Z))	ALL	Serum	Down	<0.05			

LysoPC (17:0)	ALL	Serum	Down	<0.05			
LysoPC (22:5(7Z,10Z,13Z,16Z,19Z))	ALL	Serum	Down	<0.05			
8,11-eicosadienoic acid	ALL	Serum	Down	<0.05			
Uric acid	ALL	Serum	Down	<0.05			
Chenodeoxycholic acid glycine conjugate	ALL	Serum	Down	<0.05			
LysoPE (22:4(7Z,10Z,13Z,16Z)/0:0)	ALL	Serum	Down	<0.05			
LysoPE (20:4(8Z,11Z,14Z,17Z)/0:0)	ALL	Serum	Down	<0.05			
PC (15:0/0:0)	ALL	Serum	Down	<0.05			
Hyaluronic Acid	BCP-ALL	Serum	Up	0.03	Latex agglutination.	Potential prognostic biomarker for adult BCP-ALL, on the day before the beginning of the chemotherapy.	[162]
SPGT	ALL	Serum	Up	<0.001	Spectrometry	Prognostic significance associated with progression fulminant form of childhood ALL.	[163]
2,3-Dinor-6-keto-PGF1a	ALL	Whole blood	High	<0.01	Mass spectrometer	Predict clinical outcome, monitor disease progress of adult ALL.	[93]
GPEtn(16:0/0:0)	ALL	Whole blood	Down	<0.01			
GPCho(O-6:0/O-6:0)	ALL	Whole blood	High	<0.01			
GPEtn(18:1(9Z)/0:0)	ALL	Whole blood	Down	<0.01			
Methyl 8-[2-(2-formyl-vinyl)-3-hydroxy-5-oxocyclopentyl]-octanoate	ALL	Whole blood	High	<0.01			
1-Tetrahexanoyl-2-(8-[3]-ladderane-octanyl)-sn-GPEtn	ALL	Whole blood	Down	<0.01		Predict clinical outcome, monitor disease progress of adult ALL.	

References for this section.

3 Ahmed, M.B.; Shehata, H.H.; Moussa, M.; Ibrahim, T.M. Prognostic significance of survivin and tumor necrosis factor-alpha in adult acute lymphoblastic leukemia. *Clin Biochem* **2012**, *45*, 112-116, doi:10.1016/j.clinbiochem.2011.08.1147.

4 Wang, D.; Lv, Y.Q.; Liu, Y.F.; Du, X.J.; Li, B. Differential protein analysis of lymphocytes between children with acute lymphoblastic leukemia and healthy children. *Leuk Lymphoma* **2013**, *54*, 381-386, doi:10.3109/10428194.2012.713104.

6 Fayed, D.; Donia, T.; El-Shanshory, M.; Ali, E.M.M.; Mohamed, T.M. Evaluation of MicroRNA92, MicroRNA638 in Acute Lymphoblastic Leukemia of Egyptian Children. *Asian Pacific journal of cancer prevention : APJCP* **2021**, *22*, 1567-1572, doi:10.31557/apjcp.2021.22.5.1567.

7 Shi, L.; Zhang, J.; Wu, P.; Feng, K.; Li, J.; Xie, Z.; Xue, P.; Cai, T.; Cui, Z.; Chen, X.; et al. Discovery and identification of potential biomarkers of pediatric acute lymphoblastic leukemia. *Proteome Sci* **2009**, *7*, 7, doi:10.1186/1477-5956-7-7.

8 Masilamani, V.; Devanesan, S.; AlSalhi, M.S.; AlQahtany, F.S.; Farhat, K.H. Fluorescence spectral detection of acute lymphoblastic leukemia (ALL) and acute myeloid leukemia (AML): A novel photodiagnosis strategy. *Photodiagnosis Photodyn Ther* **2020**, *29*, 101634, doi:10.1016/j.pdpdt.2019.101634.

9 Shahid, S.; Shahid, W.; Shaheen, J.; Akhtar, M.W.; Sadaf, S. Circulating miR-146a expression as a non-invasive predictive biomarker for acute lymphoblastic leukemia. *Scientific reports* **2021**, *11*, 22783, doi:10.1038/s41598-021-02257-4.

14 Yu, R.; Yang, S.; Liu, Y.; Zhu, Z. Identification and validation of serum autoantibodies in children with B-cell acute lymphoblastic leukemia by serological proteome analysis. *Proteome Sci* **2022**, *20*, 3, doi:10.1186/s12953-021-00184-w.

15 Swellam, M.; El-Khazragy, N. Clinical impact of circulating microRNAs as blood-based marker in childhood acute lymphoblastic leukemia. *Tumour Biol* **2016**, *37*, 10571-10576, doi:10.1007/s13277-016-4948-7.

16 Nabhan, M.; Louka, M.L.; Khairy, E.; Tash, F.; Ali-Labib, R.; El-Habashy, S. MicroRNA-181a and its target Smad 7 as potential biomarkers for tracking child acute lymphoblastic leukemia. *Gene* **2017**, *628*, 253-258, doi:10.1016/j.gene.2017.07.052.

17 Bai, Y.; Zhang, H.; Sun, X.; Sun, C.; Ren, L. Biomarker identification and pathway analysis by serum metabolomics of childhood acute lymphoblastic leukemia. *Clin Chim Acta* **2014**, *436*, 207-216, doi:10.1016/j.cca.2014.05.022.

20 Aly, R.M.; Yousef, A.B. Prognostic significance of lymphoid enhancer-binding factor-1 expression in egyptian adult B-acute lymphocytic leukemia patients. *Turk J Haematol* **2015**, *32*, 15-20, doi:10.4274/tjh.2013.0140.

21 Gutierrez-Aguirre, C.H.; Flores-Jimenez, J.A.; Alatorre-Ricardo, J.; Cantu-Rodriguez, O.G.; Rosas-Taraco, A.; Salazar-Riojas, R.; Jaime-Perez, J.C.; Sanchez-Cardenas, M.; Lopez-Silva, L.; Martinez-Castilla, A.M.; et al. The prognostic significance of serum XCL1 concentration in patients with acute lymphoblastic leukemia: a pilot study. *Ann Hematol* **2017**, *96*, 2015-2024, doi:10.1007/s00277-017-3142-3.

27 Aly, R.M.; Ghazy, H.F. Prognostic significance of MSI2 predicts unfavorable outcome in adult B-acute lymphoblastic leukemia. *Int J Lab Hematol* **2015**, *37*, 272-278, doi:10.1111/ijlh.12284.

46 Volm, M.; Hecker, S.; Sauerbrey, A.; Mattern, J. Predictive value of statin, a G0-associated cell cycle protein, in childhood acute lymphoblastic leukemia. *Int J Cancer* **1995**, *64*, 166-170, doi:10.1002/ijc.2910640304.

49 Miljkovic-Licina, M.; Arraud, N.; Zahra, A.D.; Ropraz, P.; Matthes, T. Quantification and Phenotypic Characterization of Extracellular Vesicles from Patients with Acute Myeloid and B-Cell Lymphoblastic Leukemia. *Cancers (Basel)* **2021**, *14*, doi:10.3390/cancers14010056.

50 Gao, Y.J.; He, Y.J.; Yang, Z.L.; Shao, H.Y.; Zuo, Y.; Bai, Y.; Chen, H.; Chen, X.C.; Qin, F.X.; Tan, S.; et al. Increased integrity of circulating cell-free DNA in plasma of patients with acute leukemia. *Clin Chem Lab Med* **2010**, *48*, 1651-1656, doi:10.1515/CCLM.2010.311.

62 Aref, S.; Salama, O.; Shamaa, S.; El-Refaie, M.; Mourkos, H. Angiogenesis factor pattern differs in acute lymphoblastic leukemia and chronic lymphocytic leukemia. *Hematology* **2007**, *12*, 319-324, doi:10.1080/10245330701340759.

63 Potapnev, M.P.; Petyovka, N.V.; Belevtsev, M.V.; Savitskiy, V.P.; Migal, N.V. Plasma level of tumor necrosis factor-alpha (TNF-alpha) correlates with leukocytosis and biological features of leukemic cells, but not treatment response of children with acute lymphoblastic leukemia. *Leuk Lymphoma* **2003**, *44*, 1077-1079, doi:10.1080/1042819031000068025.

64 Zakhary, N.I.; Boshra, S.A.; El-Sawalhi, M.M.; Fahim, A.T.; Ebeid, E.N. Insulin-like growth factor system in Egyptian children with acute lymphoblastic leukemia. *Genet Test Mol Biomarkers* **2012**, *16*, 1067-1072, doi:10.1089/gtmb.2012.0039.

65 Pal, S.; Bandyopadhyay, S.; Chatterjee, M.; Bhattacharya, D.K.; Minto, L.; Hall, A.G.; Mandal, C. Antibodies against 9-O-acetylated sialoglycans: a potent marker to monitor clinical status in childhood acute lymphoblastic leukemia. *Clin Biochem* **2004**, *37*, 395-403, doi:10.1016/j.clinbiochem.2004.01.001.

66 Abdel-Aziz, M.M. Clinical significance of serum p53 and epidermal growth factor receptor in patients with acute leukemia. *Asian Pacific journal of cancer prevention : APJCP* **2013**, *14*, 4295-4299, doi:10.7314/apjcp.2013.14.7.4295.

67 Pane, F.; Savoia, M.; Fortunato, G.; Camera, A.; Rotoli, B.; Salvatore, F.; Sacchetti, L. Serum pseudouridine in the diagnosis of acute leukaemias and as a novel prognostic indicator in acute lymphoblastic leukaemia. *Clin Biochem* **1993**, *26*, 513-520, doi:10.1016/0009-9120(93)80017-o.

68 Zhu, S.; Xing, C.; Li, R.; Cheng, Z.; Deng, M.; Luo, Y.; Li, H.; Zhang, G.; Sheng, Y.; Peng, H.; et al. Proteomic profiling of plasma exosomes from patients with B-cell acute lymphoblastic leukemia. *Scientific reports* **2022**, *12*, 11975, doi:10.1038/s41598-022-16282-4.

69 Elgendi, H.M.; Mekawy, M.A.; Abdel Wahab, S.E.; Tawfik, L.M.; Ismail, E.A.; Adly, A.A. AC133 expression in egyptian children with acute leukemia: impact on treatment response and disease outcome. *Journal of pediatric hematology/oncology* **2010**, *32*, 286-293, doi:10.1097/MPH.0b013e3181c80c08.

70 Mohammadi, M.; Amirmahani, F.; Goharrizi, K.J.; Pakzad, R.; Dolat, H. Evaluating the expression level of Survivin gene in different groups of B-cell acute lymphoblastic leukemia patients of Iran. *Mol Biol Rep* **2019**, *46*, 2679-2684, doi:10.1007/s11033-019-04703-z.

71 Alkhouly, N.; Shehata, I.; Ahmed, M.B.; Shehata, H.; Hassan, S.; Ibrahim, T. HLA-G expression in acute lymphoblastic leukemia: a significant prognostic tumor biomarker. *Med Oncol* **2013**, *30*, 460, doi:10.1007/s12032-013-0460-8.

72 Swellam, M.; Hashim, M.; Mahmoud, M.S.; Ramadan, A.; Hassan, N.M. Aberrant Expression of Some Circulating miRNAs in Childhood Acute Lymphoblastic Leukemia. *Biochem Genet* **2018**, *56*, 283-294, doi:10.1007/s10528-018-9844-y.

73 Luna-Aguirre, C.M.; de la Luz Martinez-Fierro, M.; Mar-Aguilar, F.; Garza-Veloz, I.; Trevino-Alvarado, V.; Rojas-Martinez, A.; Jaime-Perez, J.C.; Malagon-Santiago, G.I.; Gutierrez-Aguirre, C.H.; Gonzalez-Llano, O.; et al. Circulating microRNA expression profile in B-cell acute lymphoblastic leukemia. *Cancer Biomark* **2015**, *15*, 299-310, doi:10.3233/CBM-150465.

74 El-maadawy, E.A.; Bakry, R.M.; Moussa, M.M.; El-Naby, S.; Talaat, R.M.J.C.; Pharmacology, E.; Physiology. Alteration in miRNAs expression in paediatric acute lymphocyticleukaemia: Insight into patients' therapeutic response. **2021**, *48*, 35-43.

75 Ohyashiki, J.H.; Umezu, T.; Kobayashi, C.; Hamamura, R.S.; Tanaka, M.; Kuroda, M.; Ohyashiki, K. Impact on cell to plasma ratio of miR-92a in patients with acute leukemia: in vivo assessment of cell to plasma ratio of miR-92a. *BMC research notes* **2010**, *3*, 347, doi:10.1186/1756-0500-3-347.

76 El-Khazragy, N.; Elshimy, A.A.; Hassan, S.S.; Matbouly, S.; Safwat, G.; Zannoun, M.; Riad, R.A. Dysregulation of miR-125b predicts poor response to therapy in pediatric acute lymphoblastic leukemia. *J Cell Biochem* **2018**, doi:10.1002/jcb.28017.

77 Nemes, K.; Csoka, M.; Nagy, N.; Mark, A.; Varadi, Z.; Danko, T.; Kovacs, G.; Kopper, L.; Sebestyen, A. Expression of certain leukemia/lymphoma related microRNAs and its correlation with prognosis in childhood acute lymphoblastic leukemia. *Pathol Oncol Res* **2015**, *21*, 597-604, doi:10.1007/s12253-014-9861-z.

78 Abu Sabaa, A.; Shen, Q.; Lennmyr, E.B.; Enblad, A.P.; Gammelgard, G.; Molin, D.; Hein, A.; Freyhult, E.; Kamali-Moghaddam, M.; Hoglund, M.; et al. Plasma protein biomarker profiling reveals major differences between acute leukaemia, lymphoma patients and controls. *N Biotechnol* **2022**, *71*, 21-29, doi:10.1016/j.nbt.2022.06.005.

90 Gaffo, E.; Boldrin, E.; Dal Molin, A.; Bresolin, S.; Bonizzato, A.; Trentin, L.; Frasson, C.; Debatin, K.-M.; Meyer, L.H.; te Kronnie, G.; et al. Circular RNA differential expression in blood cell populations and exploration of circRNA deregulation in pediatric acute lymphoblastic leukemia. *Scientific reports* **2019**, *9*, 14670, doi:10.1038/s41598-019-50864-z.

93 Bannur, Z.; Teh, L.K.; Hennesy, T.; Rosli, W.R.; Mohamad, N.; Nasir, A.; Ankathil, R.; Zakaria, Z.A.; Baba, A.; Salleh, M.Z. The differential metabolite profiles of acute lymphoblastic leukaemic patients treated with 6-mercaptopurine using untargeted metabolomics approach. *Clin Biochem* **2014**, *47*, 427-431, doi:10.1016/j.clinbiochem.2014.02.013.

94 Bolkun, L.; Lemancewicz, D.; Jablonska, E.; Szumowska, A.; Bolkun-Skornicka, U.; Moniuszko, M.; Dzieciol, J.; Kloczko, J. Prognostic significance of ligands belonging to tumour necrosis factor superfamily in acute lymphoblastic leukaemia. *Leuk Res* **2015**, *39*, 290-295, doi:10.1016/j.leukres.2014.12.012.

96 El Kramani, N.; Elsherbiny, N.M.; El-Gayar, A.M.; Ebrahim, M.A.; Al-Gayyar, M.M.H. Clinical significance of the TNF-alpha receptors, TNFRSF2 and TNFRSF9, on cell migration molecules Fascin-1 and Versican in acute leukemia. *Cytokine* **2018**, *111*, 523-529, doi:10.1016/j.cyto.2018.05.025.

98 Hatzipantelis, E.S.; Athanassiou-Metaxa, M.; Gombakis, N.; Tzimouli, V.; Taparkou, A.; Sidi-Fragandrea, V.; Garipidou, V.; Papageorgiou, T.; Kleta, D.; Koliouskas, D.E.; et al. Thrombomodulin and von Willebrand factor: relation to endothelial dysfunction and disease outcome in children with acute lymphoblastic leukemia. *Acta Haematol* **2011**, *125*, 130-135, doi:10.1159/000322120.

99 Hagag, A.A.; Abdel-Lateef, A.E.; Aly, R. Prognostic value of plasma levels of thrombomodulin and von Willebrand factor in Egyptian children with acute lymphoblastic leukemia. *J Oncol Pharm Pract* **2014**, *20*, 356-361, doi:10.1177/1078155213508439.

101 El-Ghammaz, A.M.S.; Azzazi, M.O.; Mostafa, N.; Hegab, H.M.; Mahmoud, A.A. Prognostic significance of serum progranulin level in de novo adult acute lymphoblastic leukemia patients. *Clin Exp Med* **2020**, *20*, 269-276, doi:10.1007/s10238-020-00610-x.

105 Jiang, H.; Tang, J.; Qiu, L.; Zhang, Z.; Shi, S.; Xue, L.; Kui, L.; Huang, T.; Nan, W.; Zhou, B.; et al. Semaphorin 4D is a potential biomarker in pediatric leukemia and promotes leukemogenesis by activating PI3K/AKT and ERK signaling pathways. *Oncol Rep* **2021**, *45*, doi:10.3892/or.2021.7952.

106 Xue, L.; Shi, S.; Lei, H.; Zhang, Z.; Tian, X.; Qiu, L.; Tang, J.; Kui, L.; Nan, W.; Cao, X.; et al. Soluble Sema4D Level Is Positively Correlated with Sema4D Expression in PBMCs and Peripheral Blast Number in Acute Leukemia. *Dis Markers* **2022**, *2022*, 1384471, doi:10.1155/2022/1384471.

107 Yeh, C.H.; Tseng, R.; Hannah, A.; Estrov, Z.; Estey, E.; Kantarjian, H.; Albitar, M. Clinical correlation of circulating heat shock protein 70 in acute leukemia. *Leuk Res* **2010**, *34*, 605-609, doi:10.1016/j.leukres.2009.09.014.

110 Braoudaki, M.; Lambrou, G.I.; Vougas, K.; Karamolegou, K.; Tsangaris, G.T.; Tzortzatou-Stathopoulou, F. Protein biomarkers distinguish between high- and low-risk pediatric acute lymphoblastic leukemia in a tissue specific manner. *J Hematol Oncol* **2013**, *6*, 52, doi:10.1186/1756-8722-6-52.

111 Ibrahim, L.; Aladle, D.; Mansour, A.; Hammad, A.; Al Wakeel, A.A.; Abd El-Hameed, S.A. Expression and prognostic significance of livin/BIRC7 in childhood acute lymphoblastic leukemia. *Med Oncol* **2014**, *31*, 941, doi:10.1007/s12032-014-0941-4.

112 Chiaretti, S.; Brugnoletti, F.; Messina, M.; Paoloni, F.; Fedullo, A.L.; Piciocchi, A.; Elia, L.; Vitale, A.; Mauro, E.; Ferrara, F.; et al. CRLF2 overexpression identifies an unfavourable subgroup of adult B-cell precursor acute lymphoblastic leukemia lacking recurrent genetic abnormalities. *Leuk Res* **2016**, *41*, 36-42, doi:10.1016/j.leukres.2015.11.018.

115 Akpinar, F.; Polat, A.; Balci, Y.I.; Akça, H.; Şenol, H.; Tokgun, O. Microrna expression profiles and changes with treatment on childhood leukemias. **2020**.

116 Ramani, R.; Megason, G.; Schallheim, J.; Karlson, C.; Vijayakumar, V.; Vijayakumar, S.; Hicks, C. Integrative Analysis of MicroRNA-Mediated Gene Signatures and Pathways Modulating White Blood Cell Count in Childhood Acute Lymphoblastic Leukemia. *Biomark Insights* **2017**, *12*, 1177271917702895, doi:10.1177/1177271917702895.

117 Xue, Y.; Yang, X.; Hu, S.; Kang, M.; Chen, J.; Fang, Y. A genetic variant in miR-100 is a protective factor of childhood acute lymphoblastic leukemia. *Cancer medicine* **2019**, *8*, 2553-2560, doi:10.1002/cam4.2082.

118 Schotte, D.; De Menezes, R.X.; Akbari Moqadam, F.; Khankahdani, L.M.; Lange-Turenhout, E.; Chen, C.; Pieters, R.; Den Boer, M.L. MicroRNA characterize genetic diversity and drug resistance in pediatric acute lymphoblastic leukemia. *Haematologica* **2011**, *96*, 703-711, doi:10.3324/haematol.2010.026138.

119 Jain, A.; Bakhshi, S.; Thakkar, H.; Gerards, M.; Singh, A. Elevated mitochondrial DNA copy numbers in pediatric acute lymphoblastic leukemia: A potential biomarker for predicting inferior survival. *Pediatr Blood Cancer* **2018**, *65*, doi:10.1002/pbc.26874.

124 Cavalcante Mde, S.; Torres-Romero, J.C.; Lobo, M.D.; Moreno, F.B.; Bezerra, L.P.; Lima, D.S.; Matos, J.C.; Moreira Rde, A.; Monteiro-Moreira, A.C. A panel of glycoproteins as candidate biomarkers for early diagnosis and treatment evaluation of B-cell acute lymphoblastic leukemia. *Biomark Res* **2016**, *4*, 1, doi:10.1186/s40364-016-0055-6.

125 Fathi, M.; Amirghofran, Z.; Shahriari, M. Soluble Fas and Fas ligand and prognosis in children with acute lymphoblastic leukemia. *Med Oncol* **2012**, *29*, 2046-2052, doi:10.1007/s12032-011-9965-1.

126 Bruey, J.M.; Kantarjian, H.; Estrov, Z.; Zhang, Z.; Ma, W.; Albitar, F.; Abdool, A.; Thomas, D.; Yeh, C.; O'Brien, S.; et al. Circulating Ki-67 protein in plasma as a biomarker and prognostic indicator of acute lymphoblastic leukemia. *Leuk Res* **2010**, *34*, 173-176, doi:10.1016/j.leukres.2009.07.030.

127 Birgen, D.; Ertem, U.; Duru, F.; Sahin, G.; Yuksek, N.; Bozkurt, C.; Karacan, C.D.; Aksoy, C. Serum Ca 125 levels in children with acute leukemia and lymphoma. *Leuk Lymphoma* **2005**, *46*, 1177-1181, doi:10.1080/10428190500096690.

130 Pawlik-Gwozdecka, D.; Gorska-Ponikowska, M.; Adamkiewicz-Drozynska, E.; Niedzwiecki, M. Serum heat shock protein 90 as a future predictive biomarker in childhood acute lymphoblastic leukemia. *Cent Eur J Immunol* **2021**, *46*, 63-67, doi:10.5114/ceji.2020.95114.

132 Krawczuk-Rybak, M.; Leszczynska, E.; Malinowska, I.; Matysiak, M.; Ostrowska, H. Proteasome chymotrypsin-like activity in plasma as a useful marker for children with acute lymphoblastic leukemia. *Scand J Clin Lab Invest* **2012**, *72*, 67-72, doi:10.3109/00365513.2011.634021.

134 Moschovi, M.; Trimis, G.; Vounatsou, M.; Katsibardi, K.; Margeli, A.; Damianos, A.; Chrousos, G.; Papassotiriou, I. Serial plasma concentrations of adiponectin, leptin, and resistin during therapy in children with acute lymphoblastic leukemia. *Journal of pediatric hematology/oncology* **2010**, *32*, e8-13, doi:10.1097/MPH.0b013e3181b8a50c.

135 Aref, S.; Ibrahim, L.; Azmy, E.; Al Ashary, R. Impact of serum adiponectin and leptin levels in acute leukemia. *Hematology* **2013**, *18*, 198-203, doi:10.1179/1607845412y.0000000059.

137 O'Neill, K.L.; Zhang, F.; Li, H.; Fuja, D.G.; Murray, B.K. Thymidine kinase 1--a prognostic and diagnostic indicator in ALL and AML patients. *Leukemia* **2007**, *21*, 560-563, doi:10.1038/sj.leu.2404536.

139 Lopez-Martinez, B.; Vilchis Ordonez, A.; Salazar Garcia, M.; Klunder-Klunder, M.; Parra-Ortega, I.; Dorantes-Acosta, E.; Angeles-Floriano, T. Thymidine Kinase: A Biomarker for Recently Diagnosed Acute Leukemia in Pediatric Patients According to the Cell Line Involved. *Arch Med Res* **2015**, *46*, 630-634, doi:10.1016/j.arcmed.2015.11.005.

140 Yokota, A.; Ishii, G.; Sugaya, Y.; Nishimura, M.; Saito, Y.; Harigaya, K. Potential use of serum CD44 as an indicator of tumour progression in acute leukemia. *Hematological oncology* **1999**, *17*, 161-168, doi:10.1002/(sici)1099-1069(199912)17:4<161::aid-hon646>3.0.co;2-y.

141 Takeuchi, M.; Tanizawa, A.; Fukumoto, Y.; Kikawa, Y.; Mayumi, M. Serum soluble CD44 in pediatric patients with acute leukemia. *Journal of pediatric hematology/oncology* **1999**, *21*, 384-388, doi:10.1097/00043426-199909000-00009.

142 Hatzistilianou, M.; Athanassiadou, F.; Agguridaki, C.; Catriu, D. Circulating soluble adhesion molecule levels in children with acute lymphoblastic leukaemia. *Eur J Pediatr* **1997**, *156*, 537-540, doi:10.1007/s004310050657.

146 Rzepiel, A.; Kutszegi, N.; Gezsi, A.; Sagi, J.C.; Egyed, B.; Peter, G.; Butz, H.; Nyiro, G.; Muller, J.; Kovacs, G.T.; et al. Circulating microRNAs as minimal residual disease biomarkers in childhood acute lymphoblastic leukemia. *J Transl Med* **2019**, *17*, 372, doi:10.1186/s12967-019-2114-x.

150 Sun, B.; Li, L.; Xu, M.; Wang, X.; Wang, F.; Ni, H. Significance of BAFF/APRIL Expression and Their Receptors in Pediatric Patients With Acute Lymphoblastic Leukemia. *Journal of pediatric hematology/oncology* **2016**, *38*, 167-172, doi:10.1097/mph.0000000000000549.

151 Sun, B.; Wu, Y.; Wu, L.; Cui, M.; Ni, H.; Yang, Z.; Xu, M.; Wang, H. Raised expression of APRIL in Chinese children with acute lymphoblastic leukemia and its clinical implications. *Journal of pediatric hematology/oncology* **2014**, *36*, 276-280, doi:10.1097/MPH.0b013e31829f3271.

152 Meena, R.; Nangia, A.; Sharma, S.; Chandra, J. Serum Levels of Vascular Endothelial Growth Factor and Its Receptor in Newly Diagnosed Paediatric Acute Lymphoblastic Leukemia. *Indian J Hematol Blood Transfus* **2021**, *37*, 586-592, doi:10.1007/s12288-021-01413-0.

153 Olejnik, I. Serum soluble L-selectin in childhood acute lymphoblastic leukemia. *Pediatr Int* **1999**, *41*, 246-248, doi:10.1046/j.1442-200x.1999.01062.x.

154 Ebrahimi-Rad, M.; Khatami, S.; Ansari, S.; Jalylfar, S.; Valadbeigi, S.; Saghiri, R. Adenosine Deaminase 1 as a Biomarker for Diagnosis and Monitoring of Patients with Acute Lymphoblastic Leukemia. *J Med Biochem* **2018**, *37*, 128-133, doi:10.1515/jomb-2017-0042.

155 Abdool, A.; Yeh, C.H.; Kantarjian, H.; O'Brien, S.; Bruey, J.; Giles, F.; Albitar, M. Circulating CD33 and its clinical value in acute leukemia. *Exp Hematol* **2010**, *38*, 462-471, doi:10.1016/j.exphem.2010.03.016.

156 Aref, S.; Azmy, E.; El-Bakry, K.; Ibrahim, L.; Mabed, M. Prognostic impact of CD200 and CD56 expression in adult acute lymphoblastic leukemia patients. *Hematology* **2018**, *23*, 263-270, doi:10.1080/10245332.2017.1404276.

157 Bhatia, P.; Masih, S.; Varma, N.; Bansal, D.; Trehan, A. High Expression of Lung Resistance Protein mRNA at Diagnosis Predicts Poor Early Response to Induction Chemotherapy in Childhood Acute Lymphoblastic Leukemia. *Asian Pacific journal of cancer prevention : APJCP* **2015**, *16*, 6663-6668, doi:10.7314/apjcp.2015.16.15.6663.

158 Duyu, M.; Durmaz, B.; Gunduz, C.; Vergin, C.; Yilmaz Karapinar, D.; Aksoylar, S.; Kavakli, K.; Cetingul, N.; Irken, G.; Yaman, Y.; et al. Prospective evaluation of whole genome microRNA expression profiling in childhood acute lymphoblastic leukemia. *Biomed Res Int* **2014**, *2014*, 967585, doi:10.1155/2014/967585.

159 Al Nakeeb, R.H.; Alrubaye, D.J.I.J.o.S. The Expression of Different Micrornas in Iraqi Patients with Childhood Acute Leukemia and Their Association to C/EBP-Î'Serum Level. **2020**, 2879-2887.

160 Labib, H.A.; Elantouny, N.G.; Ibrahim, N.F.; Alnagar, A.A. Upregulation of microRNA-21 is a poor prognostic marker in patients with childhood B cell acute lymphoblastic leukemia. *Hematology* **2017**, *22*, 392-397, doi:10.1080/10245332.2017.1292204.

161 Asnafi, A.A.; Khodadi, E.; Golchin, N.; Alghasi, A.; Tavakolifar, Y.; Saki, N.J.F.i.B. Association between microRNA-21, microRNA-150, and micro-RNA-451 expression and clinical outcome of patients with acute lymphoblastic leukemia. **2017**, *12*, 63-70.

162 Anagnostopoulou, E.; Papanastasopoulou, C.; Papastamataki, M.; Kotsiou, A.; Topouzoglou, Z.; Anagnostopoulos, N.; Sitaras, N. Serum Hyaluronic Acid Levels Are Altered in Acute Leukemia Patients: Potential Prognostic Implications. *Acta Haematol* **2017**, *138*, 44-51, doi:10.1159/000477574.

163 Rautonen, J.; Siimes, M.A.J.C. Elevated serum transaminase activity at diagnosis is associated with rapidly progressing disease in children with acute lymphoblastic leukemia. **1988**, *61*, 754-757.
